# COVID-19 perturbation on US air quality and human health impact assessment

**DOI:** 10.1093/pnasnexus/pgad483

**Published:** 2024-01-02

**Authors:** Jian He, Colin Harkins, Katelyn O’Dell, Meng Li, Colby Francoeur, Kenneth C Aikin, Susan Anenberg, Barry Baker, Steven S Brown, Matthew M Coggon, Gregory J Frost, Jessica B Gilman, Shobha Kondragunta, Aaron Lamplugh, Congmeng Lyu, Zachary Moon, Bradley R Pierce, Rebecca H Schwantes, Chelsea E Stockwell, Carsten Warneke, Kai Yang, Caroline R Nowlan, Gonzalo González Abad, Brian C McDonald

**Affiliations:** Cooperative Institute for Research in Environmental Sciences, University of Colorado Boulder, Boulder, CO 80309, USA; NOAA Chemical Sciences Laboratory, Boulder, CO 80305, USA; Cooperative Institute for Research in Environmental Sciences, University of Colorado Boulder, Boulder, CO 80309, USA; NOAA Chemical Sciences Laboratory, Boulder, CO 80305, USA; Department of Environmental and Occupational Health, Milken Institute School of Public Health, George Washington University, Washington, DC 20052, USA; Cooperative Institute for Research in Environmental Sciences, University of Colorado Boulder, Boulder, CO 80309, USA; NOAA Chemical Sciences Laboratory, Boulder, CO 80305, USA; Cooperative Institute for Research in Environmental Sciences, University of Colorado Boulder, Boulder, CO 80309, USA; NOAA Chemical Sciences Laboratory, Boulder, CO 80305, USA; Department of Mechanical Engineering, University of Colorado Boulder, Boulder, CO 80309, USA; Cooperative Institute for Research in Environmental Sciences, University of Colorado Boulder, Boulder, CO 80309, USA; NOAA Chemical Sciences Laboratory, Boulder, CO 80305, USA; Department of Environmental and Occupational Health, Milken Institute School of Public Health, George Washington University, Washington, DC 20052, USA; NOAA Air Resources Laboratory, College Park, MD 20740, USA; NOAA Chemical Sciences Laboratory, Boulder, CO 80305, USA; NOAA Chemical Sciences Laboratory, Boulder, CO 80305, USA; NOAA Chemical Sciences Laboratory, Boulder, CO 80305, USA; NOAA Chemical Sciences Laboratory, Boulder, CO 80305, USA; NOAA National Environmental Satellite, Data, and Information Service, Center for Satellite Applications and Research, College Park, MD 20740, USA; Cooperative Institute for Research in Environmental Sciences, University of Colorado Boulder, Boulder, CO 80309, USA; NOAA Chemical Sciences Laboratory, Boulder, CO 80305, USA; Cooperative Institute for Research in Environmental Sciences, University of Colorado Boulder, Boulder, CO 80309, USA; NOAA Chemical Sciences Laboratory, Boulder, CO 80305, USA; NOAA Air Resources Laboratory, College Park, MD 20740, USA; Earth Resources Technology (ERT) Inc., Laurel, MD 20707, USA; Space Science and Engineering Center, University of Wisconsin-Madison, Madison, WI 53706, USA; NOAA Chemical Sciences Laboratory, Boulder, CO 80305, USA; Cooperative Institute for Research in Environmental Sciences, University of Colorado Boulder, Boulder, CO 80309, USA; NOAA Chemical Sciences Laboratory, Boulder, CO 80305, USA; NOAA Chemical Sciences Laboratory, Boulder, CO 80305, USA; Department of Atmospheric and Oceanic Science, University of Maryland, College Park, MD 20742, USA; Center for Astrophysics, Harvard and Smithsonian, Cambridge, MA 02138, USA; Center for Astrophysics, Harvard and Smithsonian, Cambridge, MA 02138, USA; NOAA Chemical Sciences Laboratory, Boulder, CO 80305, USA

**Keywords:** emission inventory, air quality, health impacts, COVID-19

## Abstract

The COVID-19 stay-at-home orders issued in the United States caused significant reductions in traffic and economic activities. To understand the pandemic's perturbations on US emissions and impacts on urban air quality, we developed near-real-time bottom-up emission inventories based on publicly available energy and economic datasets, simulated the emission changes in a chemical transport model, and evaluated air quality impacts against various observations. The COVID-19 pandemic affected US emissions across broad-based energy and economic sectors and the impacts persisted to 2021. Compared with 2019 business-as-usual emission scenario, COVID-19 perturbations resulted in annual decreases of 10–15% in emissions of ozone (O_3_) and fine particle (PM_2.5_) gas-phase precursors, which are about two to four times larger than long-term annual trends during 2010–2019. While significant COVID-induced reductions in transportation and industrial activities, particularly in April–June 2020, resulted in overall national decreases in air pollutants, meteorological variability across the nation led to local increases or decreases of air pollutants, and mixed air quality changes across the United States between 2019 and 2020. Over a full year (April 2020 to March 2021), COVID-induced emission reductions led to 3–4% decreases in national population-weighted annual fourth maximum of daily maximum 8-h average O_3_ and annual PM_2.5_. Assuming these emission reductions could be maintained in the future, the result would be a 4–5% decrease in premature mortality attributable to ambient air pollution, suggesting that continued efforts to mitigate gaseous pollutants from anthropogenic sources can further protect human health from air pollution in the future.

**Significance Statement**

Near-real-time US emission inventories developed in this work are able to track emission changes from key anthropogenic source sectors during the COVID-19 pandemic lockdown and rebounding periods. COVID-induced emission reductions persist to summer 2021 especially for nitrogen oxides, due to the continuous pandemic impacts on traffic and economic activities despite the stay-at-home orders being lifted. Reducing ozone and aerosol precursor emissions annually by 10–15% led to 3–4% decreases in annual fourth maximum of daily maximum 8-h average ozone and annual fine particles and therefore a 4–5% decrease in premature mortality attributable to ambient air pollution. Long-term sustained efforts to control ozone and aerosol precursor emissions across broad-based energy and economic sectors can lessen health impacts in the future.

## Introduction

The outbreak of COVID-19 provides an unprecedented opportunity to assess changes in anthropogenic emissions and urban air quality. A range of lockdown measures were implemented in different countries and regions to suppress the local transmission of COVID-19, resulting in significant reductions in traffic and economic activities (1, 2). The subsequent lockdown impacts on air quality have been assessed over many countries and regions (3–12), showing large heterogeneity due to different levels of stringency in measures (i.e. stringency indexes) aimed at reducing spread of the virus (5, 13). Most such studies focus on the observational analysis of air pollutants and fewer on air quality modeling (5), mainly due to the lack of up-to-date bottom-up emission inventories under the lockdown scenario, which often take multiple years to create.

Observation-based top-down estimates of COVID-induced emission changes largely rely on data availability. As a result, most top-down estimates have focused on nitrogen dioxide (NO_2_) (2, 6, 10, 14–16), with fewer studies on volatile organic compounds (VOCs) (17, 18). Both are important precursors for ozone (O_3_) and fine particles (PM_2.5_). There have also been efforts to develop bottom-up emission inventories accounting for the pandemic perturbations, but mostly at the country level (19–21). A systematic quantification of emission changes for major species with a high level of detail (e.g. spatial and temporal variability, sectoral information) is critical for better understanding the drivers of air quality changes especially at a regional scale. In addition, uncertainties remain in quantifying air quality impacts induced by these emission changes (5) that can be only partly addressed by applying meteorological corrections to observations (3, 22–24). Chemical transport models are needed to attribute air quality changes to various emissions and meteorological drivers at a larger scale. However, a lack of comprehensive emission inputs that account for rapid changes in human activity due to the COVID-19 pandemic with high confidence complicates the interpretation of modeling results.

Air pollution exposure has numerous adverse health impacts and has been linked to premature mortality (25–28). Several studies have been conducted to assess the health impacts of short-term exposure to air pollutants during the COVID-19 lockdown periods (12, 29–32). However, the chronic effects of air pollution on human health are significantly larger than for acute exposure. The potential long-term health benefits of similar future, sustainable emissions reductions have yet to be assessed.

In this study, we have developed comprehensive bottom-up emission inventories over the United States capable of near-real-time (NRT) emission adjustments (1- to 3-month lag). We simulated the COVID-19 perturbations on US emissions in a chemical transport model to link emissions to air quality impacts. We evaluated the COVID-19 lockdown impact on key emission sectors and modeled air quality with various observations. In addition, we disentangled air quality changes between emission changes and meteorological variability. Lastly, we assessed the human health impacts due to changes in anthropogenic emissions, to better understand implications for future mitigation of air pollution over the contiguous United States.

## Results and discussion

### NRT emission inventories

**Fig. 1.**
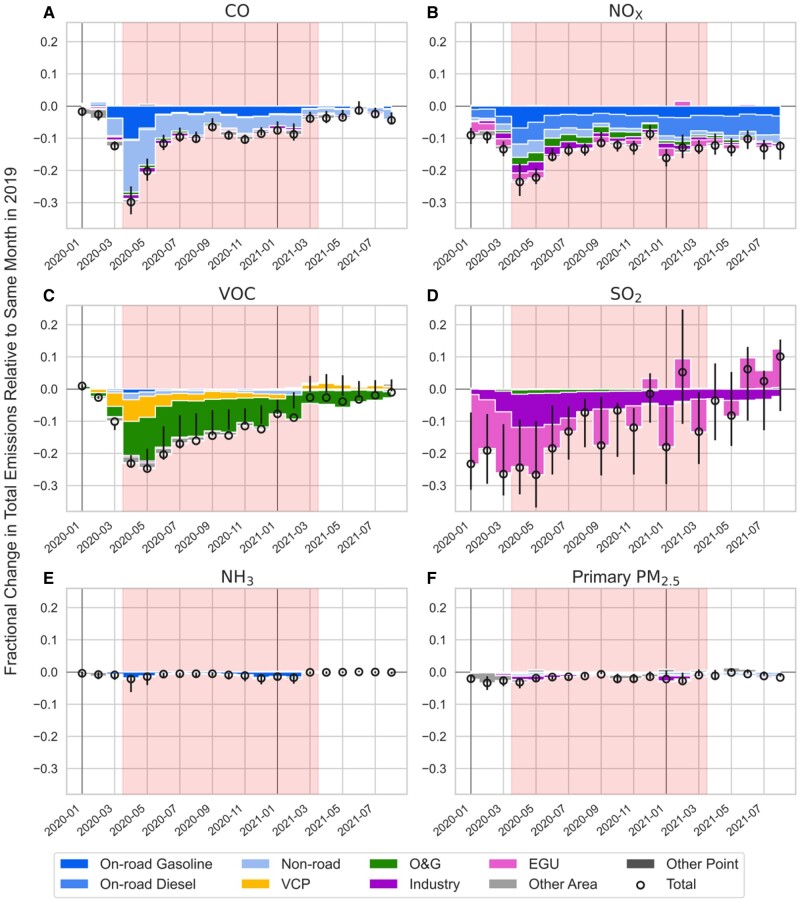
Fractional emission changes of major species relative to 2019 by sectors: A) CO; B) NO*_x_*; C) VOCs; D) SO_2_; E) NH_3_; F) primary fine particulate matter (PM_2.5_). Shaded areas cover the period of April 2020 to March 2021 for the health impact assessment. The lower and upper limits of the error bars represent 20th and 80th percentile state-level changes with the circle representing US total changes. Note, decreases in O&G emissions arise from a few highly emitting states, and fall below the 20th percentile of state variability trends but drive the overall national VOC trend.

Figure 1 shows monthly emissions for major air pollutant species relative to 2019 broken down by emission sector. In 2020, the emission decreases of carbon monoxide (CO) and nitrogen oxides (NO*_x_*) were dominated by decreases in mobile sources. Specifically, emissions from nonroad engines dominated total CO emission changes, whereas emissions from on-road gasoline vehicles and diesel vehicles dominated total NO*_x_* emission changes. The largest decrease in mobile sources occurred in April and May 2020, due to the largest drop in gasoline consumption (by about 40%) in April and a significant drop in diesel consumption during April–June (SI Appendix, Fig. S1A). National VOC emission changes were driven by changes in the oil and gas (O&G) sector, where the largest drop in wholesale petroleum production occurred in May 2020 (SI Appendix, Fig. S1E). VOC emissions from the O&G sector are sensitive to O&G production as estimated in the fuel-based O&G inventory (33). Over urban source regions, VOC emission changes were dominated by changes from volatile chemical products (VCPs), with the largest reduction in April 2020. By the end of 2020, changes in VCP emissions were small. Point sources (including industrial and power plant sources) dominated the emission changes of sulfur dioxide (SO_2_), with the largest reductions in March–May 2020. The large month-to-month variability in SO_2_ emissions is mainly due to electricity generation units with stack monitors reported through the Continuous Emissions Monitoring Systems (CEMS; SI Appendix, Fig. S2A and B). The overall changes in ammonia (NH_3_) and primary PM_2.5_ emissions are small (within 2%), as we assume no changes in agricultural NH_3_ emissions and fugitive dust emissions due to the lack of specific activity data. However, we do not expect large COVID-19 impacts on agricultural activities as similar assumptions were investigated over Europe (34). Over the urban areas, the major reductions in NH_3_ emissions were due to the decreases in mobile sources, by about 10% during the lockdown period. While total annual emissions generally decrease from 2019 to 2020, sectoral contributions do not change much between 2019 and 2020 (SI Appendix, Fig. S3).

In summary, the COVID-19 perturbations resulted in decreases in US NO*_x_* and VOC emissions by 20 ± 3 and 19 ± 7% (US average ± 1σ, state variability), respectively, averaged over April–June. The estimated COVID-induced NO*_x_* emission reductions during the lockdown months in this work are comparable with previous top-down or bottom-up estimates (16, 19, 22), but VOC emission reductions are slightly smaller compared with other bottom-up estimates (19), which could be partly due to the different adjustments in VCP emissions (SI Appendix, Supplementary Text). With the stay-at-home orders being lifted, emissions started to rebound, however, were still lower through the rest of 2020 to spring 2021 compared with the same months in 2019 (Fig. 1). By summer 2021, emissions have mostly rebounded except for NO*_x_* emissions, which were still lower than emissions in summer 2019 by about 10%, mainly due to the continuous COVID impacts on economic and traffic activities. Compared with a business-as-usual emissions scenario, COVID-induced emission perturbations led to overall annual decreases in US CO, NO*_x_*, VOC, and SO_2_ emissions by 11 ± 2, 14 ± 2, 13 ± 5, and 12 ± 10%, respectively, with small reductions in NH_3_ and primary PM_2.5_ emissions (<2%) during April 2020 to March 2021. The annual emission changes of CO, NO*_x_*, and VOC due to COVID-19 perturbations are significantly larger than the annual emission trends during 2010–2019 (−3, −6, and −2% per year, respectively) reported by the US Environmental Protection Agency (EPA, 35), whereas SO_2_ and PM_2.5_ emission changes are comparable with the annual emission trends (−14 and −2% per year), and NH_3_ emission changes are in the opposite direction to the long-term trend (+2% per year).

### Atmospheric evaluation of emission sectors

**Fig. 2.**
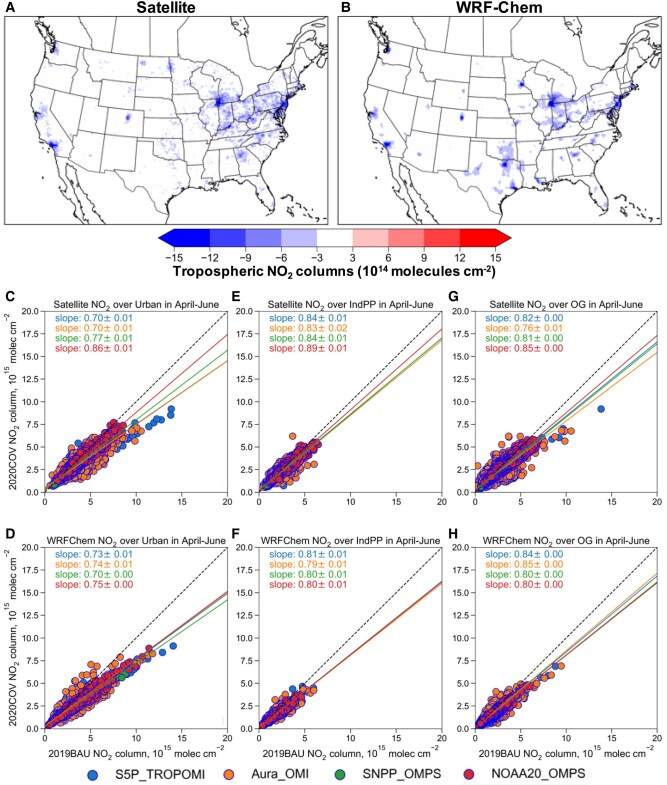
Evaluation of simulated tropospheric NO_2_ column concentrations with multiple satellite observations during between April–June 2019 and 2020. A) Observed NO_2_ changes based on the average of four satellite instruments (S5P TROPOMI, Aura OMI, S-NPP OMPS, and NOAA-20 OMPS) with air mass factors or shape factors in the satellite products replaced by the model profiles. B) Model simulated NO_2_ changes based on the average of resampled model data along each satellite track. C–H) NO_2_ columns over urban (C and D), industrial/power plant (E and F), and O&G (G and H) source regions from satellite data (C, E, and G) and model estimates (D, F, and H) for 2019BAU (*x*-axis) and 2020 COVID scenario (2020COV, *y*-axis). Slope is calculated based on the orthogonal distance regression with 95% CI.

We focus on tropospheric NO_2_ column changes to validate activity changes in transportation, industrial, and O&G sectors during April to June when anthropogenic emission changes were significant while fire impacts were minor for 2019, 2020, and 2021 during these months (36, SI Appendix, Supplementary Text, Figs. S4 and S5). We consider 2019 emissions as the business-as-usual emission scenario (hereinafter referred to as BAU), 2020 emissions as COVID-induced emission reduction scenario (hereinafter referred to as COV), and 2021 emissions as rebounded emission scenario (hereinafter referred to as REB). We evaluate tropospheric NO_2_ column changes with satellite observations, as they are helpful to detect and distinguish emission changes over urban, point sources, and O&G regions (15, 37). With our developed emission inventories, our model is able to capture satellite observed decreases in tropospheric NO_2_ column concentrations across the United States between April–June 2019 and 2020 (Fig. 2A and B). Specifically, multiple satellite observations suggest 14–30% decreases in tropospheric NO_2_ column concentrations over urban source regions (Fig. 2C), where the transportation sector dominates NO*_x_* emissions. Decreases of 11–17% in NO_2_ columns were observed over industrial and power plant source regions (Fig. 2E), and decreases of 15–24% in NO_2_ columns were observed over O&G production regions from reduced engine activity associated with drilling and extraction (Fig. 2G). These changes are well captured by the model with an overall model-satellite discrepancy within 10% (Fig. 2D and F). The decreases in NO_2_ columns are largely resulted from COVID-19-induced emission reductions with overall meteorological impacts within 2% over these emission source regions (SI Appendix, Fig. S6), suggesting these sectors are well adjusted in our emission inventories. Similarly, with the rebounded emissions in 2021, our NRT inventory and model are also able to capture the observed increases in NO_2_ columns over urban, industrial/power plant, and O&G source regions (SI Appendix, Fig. S7), demonstrating our capability to generate up-to-date emissions.

For VOCs, we compare our bottom-up estimates of VCP emission changes with ambient measurements at a ground site in Boulder, CO, USA (SI Appendix, Supplementary Text). The measurements suggest that the emissions of D5-Siloxane, a personal care product tracer, decreased by 50% in April–May 2020 relative to February 2018, and started to increase in June 2020, which shows a similar trend as emissions of benzene, a mobile source tracer (SI Appendix, Fig. S8A). In contrast, the emissions of parachlorobenzotrifluoride, a tracer for solvent-based coatings, doubled in April 2020 relative to February 2018 with no significant monthly variability afterwards (SI Appendix, Fig. S8B). The emission trends of these individual VCP sectors are consistent with declining retail sales of Health and Personal Care stores and increasing sales of Building Material stores (SI Appendix, Fig. S8C), further demonstrating the consistency of our urban VOC emission inventory with atmospheric observations. Over O&G regions, it has been demonstrated that NO*_x_* emissions from engine activity for drilling and production processes are well correlated with methane and nonmethane VOC emissions (33, 38). This suggests that the O&G inventory is able to capture changes in NO*_x_* emissions in O&G fields due to the pandemic, which provides a first-order inference on how VOC emissions likely changed in this sector. In addition, we conduct satellite evaluations for tropospheric formaldehyde (HCHO) columns using similar approaches as for tropospheric NO_2_ columns (SI Appendix, Fig. S9). Despite the spatial heterogeneity in the HCHO changes across various satellite products, mainly due to the uncertainties in the retrieved slant column densities, air mass factors, and reference sector corrections (39), our model with developed emission inventories is generally able to capture observed HCHO column reductions between 2019 and 2020, with an overall model-satellite discrepancy within 10% over urban, point, and O&G source regions.

### Air quality impacts evaluation and attribution

**Fig. 3.**
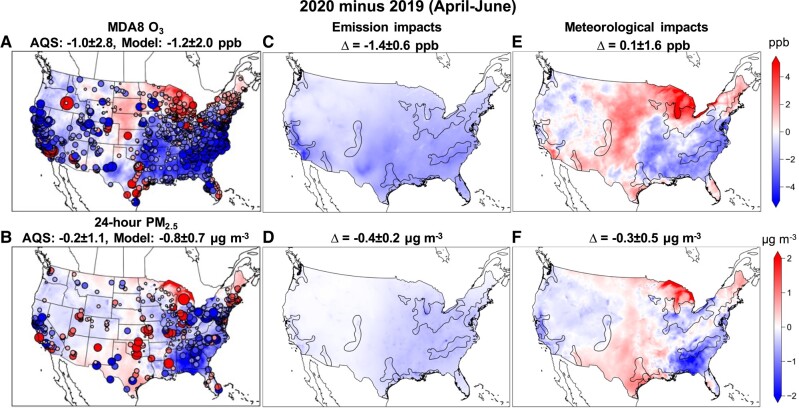
April–June changes in MDA8 O_3_ (upper panels, A, C, and E) and 24-h averaged PM_2.5_ (lower panels, B, D, and F) from 2019 to 2020. A and B) Circles overlaid on model simulated air quality changes represent observed changes from AQS surface monitoring sites with size in proportion to the absolute changes; site-averaged changes ± SD from AQS observations and model estimates are shown above each figure. C–F) Air quality impacts due to emission changes only (C and D) and due to meteorological variability only (E and F); groups of metropolitan areas are shown in black polylines; population-weighted averaged changes ± SD from model grids are shown above each figure.

We also look at the overall model performance in simulating key air pollutant concentrations. Our model is generally able to reproduce observed air quality over the United States for 2019, 2020, and 2021, respectively (SI Appendix, Table S4). Comparing April–June 2020 with 2019, surface observations from US EPA Air Quality System (AQS) show changes in MDA8 O_3_ varying from −12.3 to 17.3 ppb across the United States, with an averaged change of −1.0 ± 2.8 ppb based on all the AQS sites (Fig. 3A). While there is an overall reduction in MDA8 O_3_ across the United States from 2019 to 2020, there are noticeable increases in MDA8 O_3_ over the Great Lakes, Los Angeles, and Texas metropolitan areas. Our model is generally able to capture these changes (for about 75% of total AQS sites) despite small low biases over a few urban sites. With the emissions rebounding in 2021, MDA8 O_3_ also increases across most of the US metropolitan areas (except Texas and Gulf Coast) as observed by AQS sites with an average of 0.9 ± 2.7 ppb, which is also generally captured in our model (SI Appendix, Fig. S10A). The observed changes in 24-h PM_2.5_ vary from −5.4 to 4.5 μg m^−3^, with an averaged change of −0.2 ± 1.1 μg m^−3^ during April–June 2020 relative to 2019 (Fig. 3B), and an averaged change of 0.8 ± 1.2 μg m^−3^ during the same period in 2021 relative to 2020, which are also generally captured by the model (SI Appendix, Fig. S10B). We acknowledge model uncertainties associated with the coarse spatial resolution of the contiguous model (12 km × 12 km) and process-level representations, which add uncertainty to simulating ozone chemistry and PM_2.5_ for individual localities. Missing fire emissions, despite being small during April–June, could also contribute to the model bias in estimating local O_3_ and PM_2.5_ concentrations. However, inclusion of fire emissions may not necessarily improve model performance due to uncertainties associated with fire emissions and model representations of plume rise (SI Appendix, Fig. S5, and Table S5). Nevertheless, on the broader scale, the model demonstrates reasonably good skill in air quality simulations at regional and continental scales.

As shown in Fig. 3A and B, the overall impacts on air pollutants show large spatial heterogeneity despite relatively consistent emission changes across the United States (Fig. 3C and D). There are general decreases in MDA8 O_3_ and 24-h PM_2.5_ across groups of metropolitan areas (shown in black polyline in Fig. 3C and D) due to COVID-induced emission reductions during April–June 2020 compared with the same period in 2019. MDA8 O_3_ decreases by a population-weighted average of 1.4 ppb (or by 3%), with noticeably larger decreases over Southern California (−1.7 ± 0.8 ppb). Jiang et al. (40) showed emission reductions led to small increases in MDA8 O_3_ over urban areas in Southern California during the lockdown period, which is mainly due to the impacts of emission reductions on O_3_ production regime (e.g. reductions in NO*_x_* emissions over VOC-limited regime). Similar impacts on surface ozone over VOC-limited regimes are also found in China and Europe (30, 41, 42). Interestingly, Schroeder et al. (43) showed the COVID-induced NO*_x_* reductions were sufficient to shift the O_3_ production in the South Coast Air Basin into the NO*_x_*-limited regime. Our inventory suggests urban NO*_x_* and VOC emissions reduced by 22 ± 5 and 16 ± 5% during the lockdown period (April–June). The satellite-retrieved ratio of HCHO to NO_2_ (FNR), used to indicate O_3_ formation chemistry, also shows increases over urban source regions during April–June 2020 compared with the same period in 2019 (SI Appendix, Fig. S11), which is well captured in our model. If we use the FNR derived by Jin et al. (44) as the indicator for the transition regime over Los Angeles (4.1 to 5.0), FNR estimated in this work also shows Los Angeles shifts toward transition regime with some areas into the NO*_x_*-limited regime. The impact on O_3_ formation chemistry over Los Angeles is more consistent with Schroeder et al. (43). There is not significant spatial heterogeneity in the emission-induced PM_2.5_ decrease (by a population-weighted average of 0.4 μg m^−3^ or by 5%), dominated by reductions in secondary organic aerosol (SOA, 28%), sulfate (SO_4_, 25%), primary organic aerosols (POAs, 18%), and ammonium (NH_4_, 11%). The decreases in PM_2.5_ components are mainly due to the decreases in the precursor emissions (e.g. VOC, SO_2_, and NH_3_), primary aerosol emissions (e.g. POA), as well as impacts on oxidation chemistry or thermodynamics induced by emission reductions for secondary aerosol formation (e.g. SOA and NH_4_). Over Southern California, emission reductions lead to decreases in population-weighted PM_2.5_ by 0.6 ± 0.2 μg m^−3^ in April 2020, comparable to the estimates in Jiang et al. (40). However, the overall impacts over the United States are considerably smaller compared with those estimated in other countries and regions (3, 12). For example, Giani et al. (30) estimated the impacts of lockdown to lower PM_2.5_ by 14.5 μg m^−3^ over China and 2.2 μg m^−3^ over Europe, and Menut et al. (8) estimated a reduction in PM_2.5_ by 5–10% over the Europe. The smaller impacts on PM_2.5_ over the United States are possibly in part due to less stringent lockdown measures implemented in the United States (13) and small contributions of primary PM_2.5_ emissions from the transportation sector. Emission-induced changes in O_3_ and PM_2.5_ from 2020 to 2021 generally move in the opposite direction (SI Appendix, Fig. S10C and D) compared with the changes from 2019 to 2020.

To assess annual emission impacts, we also extended the controlled simulations (driven by same meteorology) from April 2020 to March 2021 under BAU and COV emission scenarios separately. With continuous emission reductions during April 2020 to March 2021, urban VOC/NO*_x_* ratios tends to increase due to larger reduction in urban NO*_x_* emissions (by an annual average of 16 ± 4%) than VOC emissions (by an annual average of 9 ± 3%), which could affect O_3_ production suggested by the indicator ratios (45, 46). Although such ratios indicating the transition regimes (e.g. 3.0–4.5) may vary across different megacities (44), there is a noticeable increase in FNR by 11–20% over United States major cities due to the significant emission reductions during the lockdown period (SI Appendix, Fig. S11), with an overall annual increase of 10% over urban source regions (SI Appendix, Fig. S12A). This suggests that under the COVID emission reducing scenario, O_3_ formation over VOC-limited urban areas tends to shift toward transition or NO*_x_*-limited regimes and O_3_ formation over NO*_x_*-limited urban areas becomes more sensitive to NO*_x_* emissions. Therefore, controlling NO*_x_* emissions would help lower annual O_3_ concentrations over most of the urban regions in the United States. Decreases in NO*_x_* emissions also suggest lower hydroxyl radicals (SI Appendix, Fig. S12B) through photolysis and secondary oxidation, resulting in lower atmospheric oxidation capacity. Interestingly, the fifth percentile of hourly O_3_ over urban areas slightly increases (SI Appendix, Fig. S12C), likely due to reduced nighttime O_3_ titration as also found in other studies (47). Meanwhile, the fourth highest MDA8 O_3_ (during April 2020 to March 2021) decreases by a population-weighted average of −3.1 ± 1.1 ppb (or by 4%) compared with the business as usual emission scenario (SI Appendix, Fig. S12D), which is about three times higher than the average annual O_3_ trend (0.8 ppb per year) during 2010–2019 reported from the air quality monitoring network (48). Besides the reductions in primary PM_2.5_ and its precursor emissions, the decreased atmospheric oxidation capacity also leads to decreases in secondary aerosol formation. Therefore, controlling O_3_ precursor emissions (e.g. NO*_x_* and VOC) could also reduce secondary formation of PM_2.5_. The decreases in annual total PM_2.5_ concentrations are dominated by the decrease in SOA (25%), POA (21%), SO_4_ (21%), and elemental carbon (EC, 12%; SI Appendix, Fig. S13A–D). The SOA formation represented in the model is based on a volatility basis-set mechanism, with different SOA yields depending on high or low NO*_x_* conditions (49). With comparable reductions in both NO*_x_* and VOC emissions (by an annual average of 14%), population-weighted anthropogenic SOA decreases by 0.04 μg m^−3^ (or by 3.3%) and biogenic SOA decreases by 0.03 μg m^−3^ (or by 1.5%) despite biogenic VOC emissions remain the same in BAU and COV case. Meanwhile, with a 12% decrease in annual SO_2_ emissions, population-weighted SO_4_ decreases by 0.06 μg m^−3^ (or by 5%). Controlling SO_4_ precursor emissions is more efficient than SOA anthropogenic precursor emissions as the latter is also affected by nonlinear oxidation chemistry due to the emission reductions. In addition, the annual decrease of population-weighted nitrate (NO_3_) and NH_4_ are about 10 and 6%, respectively (SI Appendix, Fig. S13E and F), contributing to 10 and 11% of total PM_2.5_ reductions. The decreases in NO_3_ and NH_4_ are mainly due to the reductions in the precursor emissions and impacts on thermodynamics induced by emission reductions. The PM_2.5_ composition does not change much between BAU and COV cases, with SOA (32%), POA (22%), primary unspeciated PM_2.5_ (22%) and SO_4_ (12%) as major PM_2.5_ components, and NH_4_ (5%), EC (4%), and NO_3_ (3%) with smaller contributions to total PM_2.5_. Due to the overall emission reductions, population-weighted PM_2.5_ decreases during April 2020 to March 2021, by an annual average of −0.3 ± 0.1 μg m^−3^ (or by 3%), which is comparable with the average annual PM_2.5_ trend during 2010–2019 reported by the US EPA (50). This suggests additional efforts are needed to understand sources of PM_2.5_ and its components so as to better control emissions of primary PM_2.5_ and its precursors (e.g. VOC, SO_2_, and NH_3_), including from agriculture.

Unlike the relatively consistent emissions impacts on air quality across the United States, meteorological variability results in much larger spatial heterogeneity of air quality responses (Fig. 3E and F). Compared with April–June 2019, regional averaged MDA8 O_3_ and 24-h PM_2.5_ vary from −2.3 to +1.9 ppb and from −1.0 to +0.6 μg m^−3^ across groups of metropolitan areas. Synoptic meteorological conditions play important roles in affecting regional air pollutant concentrations (51, 52). For example, higher temperature increases biogenic emissions and secondary formation of air pollutants, whereas stagnation associated with slower mixing often results in poorer air quality. Compared with April–June 2019, meteorological conditions in 2020 tend to increase MDA8 O_3_ or PM_2.5_ over several metropolitan areas such as Southern California, Arizona, and the Colorado Front Range, where the meteorology induced negative air quality impacts (increases in concentrations) are mostly compensated by the anthropogenic emission-induced positive impacts (decreases in concentrations). On the contrary, meteorological impacts dominate over the emission impacts on O_3_ over the North Central Plains states and PM_2.5_ changes over the South Central Plains states. At the same time, meteorology also tends to decrease MDA8 O_3_ or PM_2.5_ over other metropolitan areas (e.g. Atlanta), amplifying the positive air quality impacts induced by emission reductions. As a result, there are mixed responses in air quality across the contiguous United States. The spatial heterogeneity in the air quality responses resulted from meteorological variability also stands for the comparison of 2020 to 2021 (SI Appendix, Fig. S10E and F).

The controlled model simulations driven by either same emissions or meteorology directly link specific driver (e.g. meteorology or emission) to air quality changes and provides explicit estimates on the air quality impacts at a broader scale, which largely differ from previous studies through applying meteorological corrections to observations. Therefore, emission impacts on air quality estimated in this work by controlling meteorological effects are relatively smaller (especially for PM_2.5_) compared with previous estimates with meteorological corrections (3, 53). This further highlights the importance to disentangle meteorological and emission impacts on air quality in a 3D chemical transport model to better understand the implications of emission changes at regional and continental scales.

### Health impacts assessment

**Fig. 4.**
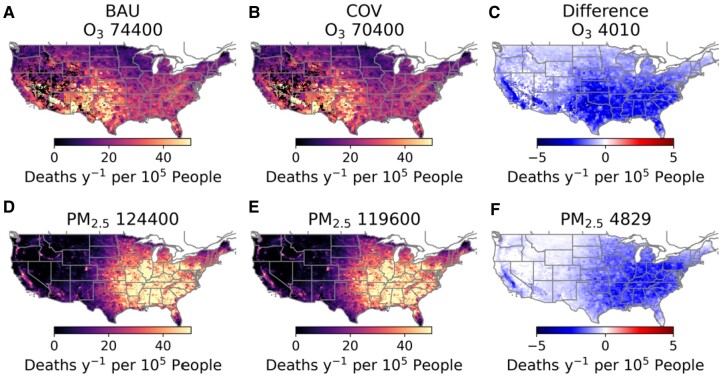
O_3_ and PM_2.5_ attributable deaths (per year per 10^5^ people) based on BAU (A and D) and COVID scenario (COV, B and E) for April 2020 to March 2021. The total attributable deaths due to each air pollutant and scenario are shown above each figure. C and F) Difference in attributable deaths between BAU and COV scenarios. The difference in the total attributable deaths are shown above each figure.

To understand the implications of long-term emission reductions on human health, we calculate O_3_ and PM_2.5_ attributable mortality for the period of April 2020 to March 2021 under BAU and COV emission scenarios. Figure 4 shows the geographic distribution of the estimated O_3_ and PM_2.5_ attributable mortality for the BAU case, COV case, and the difference between the cases, if estimated emissions reductions could be sustainably achieved for the long term. We estimate 74,400 (95% CI: 38,000–145,000) O_3_ attributable deaths per year in the BAU case, and 70,400 (95% CI: 36,000–138,000) O_3_ attributable deaths in the COV case. There are larger totals for PM_2.5_ attributable deaths, 124,400 (95% CI: 84,000–163,000) in the BAU case and 119,600 (95% CI: 81,000–157,000) in the COV case than O_3_ attributable deaths, but a similar absolute difference in attributable mortality (4,829 deaths by PM_2.5_ and 4,010 deaths by O_3_). The majority of the O_3_ attributable mortality is in the southwestern states (Fig. 4A and B), where annual mean MDA8 O_3_ concentrations are higher (SI Appendix, Fig. S14). O_3_ attributable deaths are overall lower in the COV case due to lower O_3_ concentrations (SI Appendix, Fig. S14) with larger differences over Southern California and the eastern United States (Fig. 4C). The PM_2.5_ attributable mortality and mortality differences between the BAU and COV case occur primarily in the eastern United States where annual mean 24-h PM_2.5_ concentrations are generally higher (SI Appendix, Fig. S14). The spatial pattern of differences in PM_2.5_ attributable mortality between the two cases (Fig. 4F) looks similar to that for O_3_ (Fig. 4C).

Compared with the business-as-usual emission scenario, the emissions of gas-phase precursors of O_3_ and PM_2.5_ (e.g. NO*_x_*, VOC, and SO_2_) decrease by about 10–15% in the COVID case during April 2020 to March 2021 (Fig. 1), leading to annual decreases of population-weighted MDA8 O_3_ and PM_2.5_ by 0.6 ppb (or by 2%) and 0.3 μg m^−3^ (or by 3%), respectively (SI Appendix, Fig. S14). The health impact assessment suggests if COVID-induced emission reductions could be sustained through further control of ozone and aerosol precursor emissions, an estimated 4,010 deaths could be averted per year due to reduced O_3_ exposure (5% of estimated O_3_ attributable deaths) and 4,829 deaths could be averted per year due to reduced PM_2.5_ exposure (4% of estimated PM_2.5_ attributable deaths). Comparing 2019 meteorological conditions with 2020, there are only 1% difference in population-weighted MDA8 O_3_ and <1% difference in population-weighted PM_2.5_ concentrations under the same emission scenario. The results suggest 6% of estimated O_3_ attributable deaths and 5% of estimated PM_2.5_ attributable deaths could be averted per year due to the COVID-induced emission reductions under 2019 meteorological conditions (SI Appendix, Fig. S15). This suggests there could be 1% uncertainty in the mortality calculations due to the meteorological variability when assessing emission health impacts. The COVID-induced annual emission reductions of NO*_x_* and VOC are about two to four times higher than the annual emissions trends in the preceding decade (2010–2019), which leads to a significant drop in the fourth highest MDA8 O_3_ (by −3.1 ± 1.1 ppb), while the PM_2.5_ change was in-line with long-term trends. In turn, the ozone health benefits (Fig. 4C) are of similar magnitude to PM_2.5_ (Fig. 4F). This suggests sustained reductions in ozone precursors have a meaningful impact on protecting human health, while also reducing secondary formation of PM_2.5_.

## Conclusion

In this study, we have developed NRT emission inventories for the United States to better understand the key emission sectors affected by the COVID-19 pandemic. Surface and satellite evaluation of the Weather Research and Forecasting (WRF) model coupled with Chemistry (WRF-Chem) simulations demonstrate the reliability of our emission inventory and model capabilities in simulating air quality changes. While emission changes dominate the changes in the concentrations of primary pollutants such as NO_2_ at large scale, meteorological variability plays an important role in the spatially heterogeneous impacts of secondary air pollutants such as O_3_ and PM_2.5_. COVID-induced emission perturbations result in modest decreases in annual O_3_ and PM_2.5_, leading to tangible benefits on human health. The significant changes in anthropogenic emissions due to the COVID-19 pandemic and modeling results of this study suggest that sustained efforts to control anthropogenic emission sources over long time periods can lower future concentrations of O_3_ and PM_2.5_.

## Materials and methods

### Bottom-up emission inventories

The bottom-up inventory used in this study is a hybrid of several bottom-up inventories, as well as regulatory emissions provided by US EPA through the National Emissions Inventory (NEI) 2017 (54). The bottom-up inventories include emissions from mobile source engines (fuel-based inventory of vehicle emissions, 55), VCPs (56), and O&G (fuel-based O&G, 33). To address rapid changes in human activity due to the COVID-19 pandemic, we make monthly scaling adjustments to emission sources, where data are available. These scaling adjustments are based on changes observed in relevant US energy and economic datasets and are applied to generate a NRT emission inventory. Power plant emissions are updated using CEMS data where possible (https://campd.epa.gov/). Other point and area-wide emissions are taken from the NEI 2017 and scaled with monthly adjustments developed from relevant activity metrics tracking energy consumption and economic activity in the United States. Although we use the year 2019 as the baseline emission scenario in this analysis, the 2019 emissions also vary based on the applied scalings from energy and economic datasets. By using these datasets, lockdown and prelockdown changes to economic activities have been taken into account when developing emission inventories (e.g. fuel combustion and industrial processes, SI Appendix, Figs. S1 and S2). The purpose of these NRT scaling adjustments is to generate up-to-date emissions with a minimal lag (1–3 months). The process of calculating scaling adjustments for these inventories is described in supplementary material (SI Appendix, Supplementary Text). Emissions outside of the United States for international shipping, Mexico, and Canada are from the Copernicus Atmospheric Monitoring Service Global Anthropogenic Emissions Version 4.2 (19) for the year 2019. We do not make monthly adjustments for the emissions outside the United States, as this work focuses on the understanding the impacts of emission changes within the contiguous United States.

### WRF-Chem model configurations and simulations

WRF-Chem (57) version 4.2.2 is applied to simulate emission changes and air quality impacts over the contiguous United States. The WRF-Chem model is configured at a horizontal spatial resolution of 12 km × 12 km, with total 50 vertical layers, extending from the surface to 50 hPa. Chemical boundary conditions are provided from the Realtime Air Quality Modeling System (http://raqms-ops.ssec.wisc.edu/) developed by the University of Wisconsin. A few sets of model simulations are conducted under different meteorological and emission inputs to evaluate the emission changes and to disentangle air quality impacts to emission changes and meteorological variability during April to June for 2019, 2020, and 2021. We consider 2019 emissions as BAU, 2020 emissions as COV, and 2021 emissions as REB. Paired simulation with the same anthropogenic emissions but different meteorological inputs (e.g. 2019BAU vs. 2020BAU, 2020COV vs. 2021COV) is conducted to estimate meteorological impacts. Paired simulation with the same meteorological inputs, but different anthropogenic emissions (e.g. 2020BAU vs. 2020COV, 2021COV vs. 2021REB), is conducted to estimate anthropogenic emission impacts. In addition, paired simulation for April 2020 to March 2021 with the same meteorological inputs but different anthropogenic emissions (BAU vs. COV) are conducted to assess human health impacts related to anthropogenic emission reductions, and paired simulation with the same setup but for April 2019 to March 2020 are conducted to assess meteorological variability on mortality estimates. The detailed model configurations and simulations in this study are described in the supplementary material (SI Appendix, Supplementary Text, Tables S2 and S3).

### Observations (surface + satellite) and model evaluation

We use a python-based diagnostic package MELODIES MONET (https://melodies-monet.readthedocs.io, 58) to conduct surface evaluation of MDA8 O_3_ and 24-h averaged PM_2.5_ against EPA AQS network. We evaluate simulated NO_2_ and HCHO column concentrations against multiple satellite observations provided by TROPOspheric Monitoring Instrument (TROPOMI) on board the Copernicus Sentinel-5 Precursor (S5P) satellite, Ozone Monitoring Instrument (OMI) on NASA's Aura satellite, Ozone Mapping and Profiler Suite (OMPS) on NASA/NOAA's Suomi National Polar-orbiting Partnership and NOAA-20 satellites for different source sectors. We start with level 2 satellite product and apply filtering criteria (e.g. quality flag, cloud fraction, solar zenith angle, and row anomalies) to each retrieval as recommended by the user's guides and previous work (37). The simulated NO_2_ and HCHO profiles are extracted along each valid satellite retrieval. We recalculate air mass factors in TROPOMI/OMI datasets or shape factors in OMPS datasets with those calculated from model profiles and apply averaging kernels from satellite data to the model profiles to have a fairer comparison between simulated and satellite-observed tropospheric columns (37, 59, 60). We focus on the evaluation of urban, point (industrial and powerplant), and O&G sources as they are the main sectors impacted by COVID-19 perturbations. We follow the approach described in Li et al. (37) to determine the dominance of urban, point, or O&G sources for each grid cell, which is mainly based on a fractional mobile, point, or O&G source contribution being at least 60% of the total NO*_x_* emissions under the business-as-usual condition.

### Health impacts analysis model

We apply a health impact assessment to quantify the impact of changes in annual mean MDA8 O_3_ and annual mean 24-h PM_2.5_ attributable to COVID-induced emission reductions. We use the health impact equation,


(1) Deaths=P×BR×(1−exp(−β×ΔX)),

following Anenberg et al. (61) where *P* is the population, BR is the baseline annual mortality rate, and *ΔX* is the difference between the ambient modeled pollutant concentration and the theoretical concentration at which no excess risk is assumed. For Eq. 1, *β* is defined as


(2) β=ln(RR/ΔX),

where RR is the relative risk for all-cause mortality due to long-term exposure to *ΔX*. In this work, we use relative risks for all-cause mortality from Turner et al. (62) of 1.02 (95% CI: 1.01–1.04) and 1.06 (95% CI: 1.04–1.08) for a 10 ppb increase in O_3_ and 10 µg m^−3^ in PM_2.5_, respectively. We use 2015–2019 mean county-level baseline mortality rates from the Centers for Disease Control (CDC)'s WONDER database (63) and population estimates for 2020 from the NASA SEDAC Gridded Population of the World v11.4 (64). We assume no excess risk for mortality at concentrations below 26.7 ppb for O_3_ and 2.8 µg m^−3^ for PM_2.5_, the minimum annual average exposures estimated in Turner et al. (62). All datasets are gridded to the same grid as the concentration estimates and we conduct the health impact assessment for each grid cell.

## Supplementary Material

pgad483_Supplementary_Data

## Data Availability

All the emission data are available at https://csl.noaa.gov/groups/csl7/measurements/2020covid-aqs/emissions. MELODIES MONET diagnostic package can be found at the GitHub repository https://github.com/NOAA-CSL/MELODIES-MONET. Code for health impact assessment can be found at the GitHub repository https://github.com/kaodell/COVID_HIA. S5P-PAL TROPOMI NO_2_ data are available from https://data-portal.s5p-pal.com/products/no2.html. TRPOMI HCHO data are available from Copernicus Sentinel data processed by ESA (65). OMI NO2 data are available from Krotkov et al. (66) and OMI HCHO data are available from Chance (67). OMPS NO_2_ data are provided by NOAA National Environmental Satellite, Data, and Information Service (60). OMPS HCHO data are available from Gonzalez Abad (68, 69).
