# Supplementary material for: COVID-19 perturbation on US air quality and human health impact assessment
Source: PNAS Nexus. 2024 Jan 2;3(1):pgad483. doi: 10.1093/pnasnexus/pgad483 (PMC10785034; doi:10.1093/pnasnexus/pgad483)
Supplement: pgad483_Supplementary_Data [file pgad483_supplementary_data.pdf]

Supplementary Information for

COVID-19 perturbation on US air quality and human health impact assessment

Jian He[1,2,*], Colin Harkins[1,2], Katelyn O'Dell[3], Meng Li[1,2], Colby Francoeur[1,2,4], Kenneth C. Aikin[1,2], Susan Anenberg[3], Barry Baker[5], Steven S. Brown[2], Matthew M. Coggon[2], Gregory J. Frost[2], Jessica B. Gilman[2], Shobha Kondragunta[6], Aaron Lamplugh[1,2], Congmeng Lyu[1,2], Zachary Moon[5,7], Bradley R. Pierce[8], Rebecca H. Schwantes[2], Chelsea E. Stockwell[1,2], Carsten Warneke[2], Kai Yang[9], Caroline R. Nowlan[10], Gonzalo González Abad[10], Brian C. McDonald[2]

[1]Cooperative Institute for Research in Environmental Sciences, University of Colorado Boulder, Boulder, CO 80309, USA; [2]NOAA Chemical Sciences Laboratory, Boulder, CO 80305, USA; [3]Department of Environmental and Occupational Health, Milken Institute School of Public Health, George Washington University, Washington, DC 20052, USA; [4]Department of Mechanical Engineering, University of Colorado Boulder, Boulder, CO 80309, USA; [5]NOAA Air Resources Laboratory, College Park, MD 20740, USA; [6]NOAA National Environmental Satellite, Data, and Information Service, Center for Satellite Applications and Research, College Park, MD 20740, USA; [7] Earth Resources Technology (ERT), Inc., Laurel, MD 20707, USA; [8]Space Science and Engineering Center, University of Wisconsin-Madison, Madison, WI 53706, USA; [9]Department of Atmospheric and Oceanic Science, University of Maryland, College Park, MD 20742, USA; [10]Center for Astrophysics, Harvard & Smithsonian, Cambridge, MA 02138, USA

*Corresponding author: Jian He

Email: jian.he@noaa.gov

This PDF file includes:

Supplementary text
Figures S1 to S15
Tables S1 to S5
SI References

Supplementary Information Text

Near real-time emission development

The bottom-up inventory used in this study is a hybrid of several bottom-up inventories, as well as regulatory emissions provided by US EPA through the National Emissions Inventory (NEI) 2017. The bottom-up inventories, include emissions from mobile source engines (**F**uel-based **I**nventory of **V**ehicle **E**missions), volatile chemical products (VCPs), and oil and gas (**F**uel-based **O**il and **G**as). Power plant emissions are updated using Continuous Emissions Monitoring System (CEMS) data where possible (https://campd.epa.gov/). Other point and areawide emissions are taken from the NEI 2017 (1) and scaled using activity metrics tracking energy consumption and economic activity. Emissions outside of the US for international shipping, Mexico, and Canada are from the Copernicus Atmospheric Monitoring Service (CAMS) Global Anthropogenic Emissions Version 4.2 (2) for the year 2019. A description of how mobile source, VCP and FOG emissions are estimated is provided below. To address rapid changes in human activity due to the COVID-19 pandemic, we make monthly scaling adjustments to emissions sources, where data is available, to generate a near real-time (NRT) emission inventory. The purpose of these NRT scaling adjustments is to generate up-to-date emissions with a minimal lag (1-3 months). Unfortunately, much of the minimal lag data used for monthly adjustments is not present at finer spatial scales than nationally. Where possible, state or regional adjustments are made (e.g., FIVE) but adjustments are predominantly at the national scale. The process of calculating scaling adjustments for these inventories is also described below. Table S1 lists the Source Classification Codes (SCC) that correspond to each emissions inventory used, as well as the data sources used to adjust individual sector emissions in near real-time.

Mobile Sources. The **F**uel-based **I**nventory of **V**ehicle **E**missions (FIVE) is utilized for mobile source engines (3, 4). Briefly, fuel sales of on-road engines are reported by state by the U.S. Federal Highway Administration. Taxable gasoline and diesel fuel sales for road transportation are downscaled from the state-level to roadways using light- and heavy-duty vehicle count data from the Highway Performance Monitoring System (https://www.fhwa.dot.gov/policyinformation/hpms.cfm), respectively. Roadway-link specific data account for ~70% of gasoline and ~80% of diesel fuel sales nationally (3). The remaining fraction of traffic is apportioned using population density as a spatial surrogate. Once fuel use is mapped, co-emitted air pollutant species can be estimated using fuel-based emission factors (e.g., g pollutant / kg fuel) derived from roadside measurements and laboratory studies. Fuel-based emissions factors have been published for light-duty gasoline and heavy-duty diesel vehicles for CO (5, 6), NO_x (4, 7, 8), VOCs (5, 9), NH_3 (10), and $PM_{2.5}$ (11). An advantage of using fuel sales for on-road activity is that regional monthly fuel sales data are available for near real-time emissions adjustments, with state-level monthly traffic estimates also available to spatially refine these adjustments to a state level (12). Once the on-road emissions have been mapped, diurnal and day-of-week activity factors for light- and heavy-duty vehicles are applied separately to estimate hourly emissions (3).

FIVE also includes emissions for non-road engines in a similar manner. Off-road distillate fuel sales are reported by state by the Energy Information Administration (https://www.eia.gov/petroleum/fueloilkerosene/) and allocated to end uses following Kean et al. (13). Non-highway use of gasoline is reported by the Federal Highway Administration (https://www.fhwa.dot.gov/policyinformation/statistics/2020/mf24.cfm). Fuel sales for off-road activity are also able to be adjusted regionally on a near real-time basis (12). Emission factors of co-emitted air pollutants (in g/kg fuel) are taken from the EPA NONROAD model (14). Non-road engine emissions are mapped spatially and temporally using surrogates from the NEI 2017 (1).

The VOC speciation profiles for gasoline and diesel engines are reported in McDonald et al. (9) and based on tunnel and laboratory studies, including profiles for liquid gasoline and headspace vapors distinct from exhaust (15). The FIVE mobile source inventory has been rigorously evaluated in previous modeling studies over Los Angeles (16), US (4), and New York City (17), and with

satellite NO_2 datasets (18). Updates due to the COVID-19 pandemic are accounted for, including rebounding of traffic after COVID-19 lockdown efforts (12). Adjustments to fuel sales are made using monthly gasoline and diesel sales from the EIA Prime Supplier Sales Volume report (https://www.eia.gov/dnav/pet/pet_cons_prim_dcu_nus_m.htm). Spatial refinement of adjustment factors, to the state-level, for some components of on-road traffic is performed using the US Federal Highway administration Traffic Volume Trends report (https://www.fhwa.dot.gov/policyinformation/travel_monitoring/tvt.cfm). Fig. S1A-B shows the ratio of mobile source gasoline and diesel consumption in 2020 relative to 2019 and 2021 relative to 2020. This shows large decreases in gasoline consumption in 2020 and strong rebounding in 2021. Diesel consumption also shows decreases in 2020 and rebounding in 2021 but the magnitude of each is much smaller than for gasoline, likely due to diesel use in goods transport and machinery.

Volatile Chemical Products. Following McDonald et al. (9), VCP emissions are estimated for coatings, inks, adhesives, personal care products, cleaning agents and pesticides. Briefly, VCP emissions were estimated by first performing a mass balance of chemical feedstocks and their distribution across a variety of products manufactured by the chemical industry. Average daily usage and VOC emission factors are reported in McDonald et al. (9) across the US. Long-term trends are taken into account using the same mass balance approach over time following Kim et al. (19). The VCP inventory reflects continuous efforts to lower the VOC content of chemical products, including architectural coatings and phasing out of solvent to waterborne formulations (20).

Nationally, around ~60% of VCP emissions are for consumer uses and ~40% for agricultural and industrial uses McDonald et al. (9). Agricultural pesticides are spatially and temporally allocated according to agricultural pesticide VOC emissions from the NEI17. Industrial uses are similarly spatially and temporally allocated according to the point source VOC inventory from the NEI17. Consumer product emissions are spatially allocated using population density. Past NOAA CSL measurements in New York City and elsewhere have shown a strong population density dependence of consumer VCP emissions (17, 21). Diurnal profiles for personal care product emissions are shown to peak in the morning and exponentially decay across the day (22). Other VCP sectors use diurnal profiles from the NEI17, which exhibit a midday peak. Detailed VOC speciation profiles were compiled in McDonald et al. (9), and updated to the latest California Air Resources Board surveys of consumer products and architectural coatings in Coggon et al. (17).

VCP emissions are adjusted using trade statistics from the US Census Bureau. Industrial VCP emissions are adjusted using monthly wholesale trade value for chemical manufacturing from the US Census Bureau (NAICS #4246, https://www.census.gov/wholesale/index.html). We adjust industrial VCP usage using wholesale chemical manufacturing production as a trends surrogate, equal to monthly *Sales* + *Change in Inventory*. Trade statistics are reported in value per month and must be adjusted to account for inflation and variation in price per amount of product. For wholesale production, we adjust the value to constant 2017 dollars using the Producer Price Index for the same North American Industry Classification System (NAICS) code, from the US Bureau of Labor Statistics (https://www.bls.gov/ppi/databases/). Finally, a rolling 3-month average is applied to the adjusted production value to reduce noise due to statistical sampling variability. Industrial VCP emissions and their data sources are further described in Table S1. Fig. S1C-D shows the ratio of wholesale chemical production in 2020 relative to 2019 and 2021 relative to 2020, which shows slight decreases throughout 2020 and a rebounding in 2021.

Consumer product VCP emissions are adjusted using monthly retail sales from the US Census Bureau (https://www.census.gov/retail/index.html) for related product types. Retail sales are used rather than wholesale production, as sales are more closely tied to consumer product VCP usage. Again, trade statistics are reported in value per month and must be adjusted to account for inflation and variation in price per amount of product. Retail sales are adjusted to constant 2017 dollars using the Urban Area Consumer Price Index less Food and Energy (https://www.bls.gov/cpi/data.htm). Finally, a rolling 3-month average (1 month to +1 month) is applied to the adjusted retail sales value to reduce statistical sampling variability. Consumer

product VCP emissions and their data sources are further described in Table S1. Fig. S1C-D also shows the two retail sales types used in the consumer VCP emission adjustments, in 2020 relative to 2019 and 2021 relative to 2020. Building materials sales increases throughout the 2020-2021 period while personal care products sales decrease sharply in 2020 and rebound sharply in 2021.

Oil & Gas. Upstream emissions from the oil and gas sector come from both the NEI17 and the FOG inventory (**F**uel-based **O**il and **G**as) (23, 24). The FOG inventory includes oil and gas emissions in production basins, for NO_x, CH_4, and non-methane VOCs. The NO_x emissions resulting from oil and gas engines (e.g., drilling rigs, compressor stations, dehydrators, etc.) are estimated using bottom-up methods and fuel statistics of energy usage by oil and gas companies that are downscaled using Enverus DrillingInfo well-level production data of oil and natural gas. Fugitive leaks of CH_4 and non-methane VOCs are estimated by ratio to NO_x using aircraft field data over oil and gas fields from the Southeast Nexus (SENEX: https://csl.noaa.gov/projects/senex/) 2013 and Shale Oil and Natural Gas Nexus (SONGNEX: https://csl.noaa.gov/projects/songnex/) 2015 studies. The emissions have been gridded nationally, and described by Francoeur et al. (24). Other co-emitted species (e.g., $PM_{2.5}$, CO, etc.) from oil and gas production regions are taken from the NEI17. Note that midstream (e.g., interstate pipelines) and downstream (e.g., refineries, fuel storage/transport facilities) oil and gas emissions are taken from the NEI17. Data sources and adjustment factor sources for oil and gas, refining and storage can be found in Table S1.

Because it is difficult to separate oil and gas production emissions between oil and natural gas components individually, since a well can produce both, we adjust upstream oil and gas emissions from the NEI using an average of monthly trends in natural gas consumption (EIA, https://www.eia.gov/totalenergy/data/monthly/) and wholesale production of petroleum (NAICS #4247, https://www.census.gov/wholesale/index.html). Wholesale petroleum production scaling factors are calculated to take into account inflation, following the same procedure as is described in the previous section. These adjustments and their average are shown in Fig. S1E, where it can be seen that the average is less responsive to fluctuations in either metric. Downstream emissions from the NEI17 are adjusted using wholesale production of petroleum from the US Census Bureau. This is because these sources primarily focus on oil refining rather than natural gas processing.

Other Anthropogenic. Other point and areawide emissions are taken from the NEI17 and adjusted with near real-time scaling factors in a similar fashion. Table S1 lists how source sectors are subset by Source Classification Codes (SCC), and the datasets used to adjust individual sectors in near real-time. For point sources, stack parameters and plume-rise are taken into account in WRF-Chem.

Electricity Generation. Emissions from electricity generation units (EGUs) are updated to include monthly facility-level Continuous Emissions Monitoring Systems (CEMS) data (https://campd.epa.gov/data) for species where emissions are available (NO_x, SO_x, CO_2). For pollutants where CEMS data is not available, monthly EIA energy consumption by fuel type for electricity generation are used to develop national scaling factors, which are taken as a 3-month rolling average. This is shown in Fig. S2A-B, where changes in electricity generation due to COVID lockdowns are not immediately apparent and trends likely reflect year-to-year differences in electricity demand resulting from weather and economic activity.

Fuel Combustion. For fuel combustion emissions in residential, industrial, and commercial settings (e.g., in boilers), national near real-time scaling factors are developed from US monthly fuel consumption data from the EIA (https://www.eia.gov/totalenergy/data/monthly/index.php) by fuel and sector (see Table S1). The breakdown by these sectors and fuel types is similar to an approach employed by Xing et al. (25), which constructed a long-term air quality inventory for model simulations. Boiler demand for commercial and residential buildings can vary greatly with weather. The national scale adjustments used here would miss the variations occurring between different regions due to weather. Currently there is not finer spatial scale monthly data available, which is a limitation of the datasets used for NRT adjustments. These fuel combustion trends are shown in Fig. S2C-D, for 2020 relative to 2019 and 2021 relative to 2020 fuel consumption. Changes are

generally small but for industrial combustion, observed reductions in 2020 and rebounding in 2021 may be related to COVID lockdown effects on economic activity.

Industrial Processes. For industrial process emissions (e.g., chemical manufacturing, paper production, etc.), near real-time scaling factors are developed from monthly wholesale trade statistics from the US Census Bureau (https://www.census.gov/wholesale/index.html). Industrial processes (differentiated by their SCC codes) are grouped by product type and are adjusted using relevant wholesale production statistics for the group. For example, industrial emissions associated with metals manufacturing and mining are scaled using inflation adjusted wholesale production of Metals & Minerals, except Petroleum (NAICS #4235). Wholesale production scaling factors are adjusted to take into account inflation using the Producer Price Index. Key wholesale production groups used for NRT adjustments to industrial process emissions are shown for 2020 relative to 2019 and 2021 relative to 2020 in Fig. S2E-H. Some sectors show evidence of reductions in 2020 and rebounding in 2021 related to COVID lockdowns impacting on economic activity (petroleum, textiles, automotive), while others do not show obvious evidence of this (food, electronics).

Rail and Shipping. Railroad emissions are adjusted using monthly carload and intermodal unit traffic from the US Bureau of Transportation Statistics (https://data.bts.gov/stories/s/m9eb-yevh). Emissions at airports (in-flight emissions are not included) are adjusted using monthly air carrier revenue miles flown from the US Bureau of Transportation Statistics (https://www.transtats.bts.gov/TRAFFIC/). Shipping emissions are adjusted using monthly cargo weight (imports + exports) of international shipping from the US Census Bureau (https://www.census.gov/data/developers/data-sets/international-trade.html). These monthly adjustments are applied uniformly at the national-scale.

Miscellaneous. Finally, for some types of emissions we do not have appropriate economic or energy statistics available to make near real-time adjustment factors. For these sectors, we rely on monthly variations from the baseline NEI17 inventory, and do not apply any year-to-year variation. These emission sources (e.g., waste disposal, agriculture, and dust) are generally not expected to vary significantly year-to-year or due to COVID-19 lockdowns.

VOC measurements at Boulder, Colorado

Volatile organic compounds were monitored in Boulder, CO during the COVID Air Quality Study (COVID-AQS). A full description of the campaign is provided by Rickly et al. (26). Briefly, gas-phase organic and inorganic compounds were monitored from the NOAA David Skaggs Research Center from March 30 - August 31, 2020. Additional measurements were performed in March 2018 at the same location. In both deployments, a proton-transfer-reaction time-of-flight mass spectrometer (PTR-ToF-MS) was deployed to measure mixing ratios of a wide range of VOCs. Here, we use PTR-ToF-MS measurements of select compounds indicative of individual emissions sectors, including D5-siloxane for personal care products (21, 22), parachlorobenzotrifluoride for architectural coatings (20, 21), and benzene for motor vehicle emissions (22). The PTR-ToF-MS was calibrated for each species using gravimetrically-prepared gas standards, or by liquid calibration as described by Coggon et al. (22).

WRF-Chem model configurations and simulations

The Weather Research and Forecasting (WRF) model coupled with Chemistry (WRF-Chem) (27) version 4.2.2 is applied to simulate emission changes and air quality impacts over the contiguous United States (CONUS). To address the research objectives outlined in the main text, the model is configured at 12 km x 12 km spatial resolution, with total 50 vertical layers that extend up to 50 hPa into the Upper Troposphere-Lower Stratosphere (UTLS). The meteorological initial and boundary conditions for the CONUS domain are from the North American Mesoscale Model (NAM, https://www.ncei.noaa.gov/products/weather-climate-models/north-american-mesoscale).

Chemical boundary conditions are provided from a global model developed by the University of Wisconsin called the Realtime Air Quality Modeling System (RAQMS, http://raqms-

ops.ssec.wisc.edu/), which includes data assimilation of satellite ozone and aerosol optical depth (AOD) products. Major physics and chemistry options utilized in the WRF-Chem setup are listed in Table S2. These settings have been well tested and evaluated previously in modeling over the Southeastern US (4), Eastern US (17), and CONUS (18). The oxygenated VOC species and their chemical reactions newly added by Coggon et al. (17) to better represent VCP emissions were added into the racm_soa_vbs scheme (i.e., chem_opt = 108) to create the RACM_ESRL_VCP mechanism. Both anthropogenic emissions and the BEIS biogenic emissions were respeciated to include the newly added oxygenated VOCs. A couple updates to isoprene chemistry were also included. The low-NO OH recycling updates included in Li et al. (18) were added, which included the isoprene hydroxy peroxy radical isomerization reaction to account for OH recycling described in McDonald et al.(4) and an update to the isoprene hydroxy peroxy radical + HO_2 reaction to the following: ISOP+HO_2=0.88 ISHP+0.12 HO+0.12 MACR+0.12 HO_2+0.12 HCHO with a reaction rate of 7.4E-13 x exp(700.0/T) where ISOP = Isoprene hydroxy peroxy radical, ISHP = isoprene hydroxy hydroperoxide, HO_2 = hydroperoxyl radical, OH = hydroxyl radical, MACR = Methacrolein, HCHO = formaldehyde, and T = Temperature. For this study, the products for the isoprene hydroxy peroxy radical + NO reaction were also updated to reflect the latest recommendations for NO_x recycling (28) to the following: ISOP+NO=0.87 MACR+0.87 NO_2+0.87 HCHO+0.87 HO_2+0.13 ISON with a reaction rate of 2.43E-12 x exp (360.0/T) where NO = nitrogen oxide, NO_2 = nitrogen dioxide, ISON = isoprene hydroxy nitrate.

To evaluate emission changes and understand air quality and associated health impacts, we have conducted several sets of model simulations listed in Table S3. Specifically, we simulate emission changes during April to June for 2019, 2020, and 2021. Each year represents different meteorological conditions. We consider 2019 emissions as business-as-usual emission scenario (BAU), 2020 emissions as COVID-induced emission reduction scenario (COV), and 2021 emissions as rebounded emission scenario (REB). Paired simulation with the same anthropogenic emissions but different meteorological inputs (e.g., 2019BAU vs 2020BAU, 2020COV vs 2021COV) are conducted to estimate meteorological impacts. Paired simulation with the same meteorological inputs but different anthropogenic emissions (e.g., 2020BAU vs 2020COV, 2021COV vs 2021REB) are conducted to estimate anthropogenic emission impacts. In addition, paired simulation for April 2020 to March 2021 with the same meteorological inputs but different anthropogenic emissions (BAU vs COV) are conducted to assess health impacts under emission reducing scenario, and paired simulation for April 2019 to March 2020 with the same meteorological inputs but different anthropogenic emissions (BAU vs COV) are conducted to assess meteorological variability impacts on mortality estimates.

Impacts of fire emissions on air quality

Warneke et al. (29) summarized the cumulative area burned in the western US for 2017-2021 (Figure 14 in Warneke's paper), which clearly shows the typical peak months for wildfire between July and October. There were no significant increases in the cumulative burned area over the western US during April-June in 2019-2021 (<3% of annual totals) but noticeable increases in the cumulative burned area started in July especially in 2021. As a matter of fact, fire impacts were well captured by TROPOMI NO_2 observations in July 2021, which were not much shown in April-June from 2019 to 2021 (Fig. S4). We therefore conducted sensitivity simulations to include fire emissions based on an earlier version of Regional ABI and VIIRS fire Emissions (RAVE) provided by NOAA's Satellite and Information Service for July 2019, 2020, and 2021 (Table S3). We see significant fire impacts over western US in July 2021 (Fig. S5). However, the simulations with inclusion of fire emissions show much larger biases of O_3 and $PM_{2.5}$ compared to the simulations without fire emissions (Table S5). We acknowledge the uncertainties associated with fire emissions and model representations of plume rise, which could partly contribute to the model biases in simulating O_3 and $PM_{2.5}$. To have fair comparisons from 2019 to 2021, we focus on the period of April-June to disentangle air quality changes due to changes in anthropogenic emissions and meteorology. As the focus of this work is to understand the impacts due to COVID-induced anthropogenic emission changes, we therefore do not include fire emissions in this work to avoid the uncertainties associated with fire emissions and fire model representations that could complicate the interpretation of modeling results. Additional work to include fire emissions and to

improve fire representations for air quality modeling could be conducted in the future to better understand fire impacts on regional air quality.

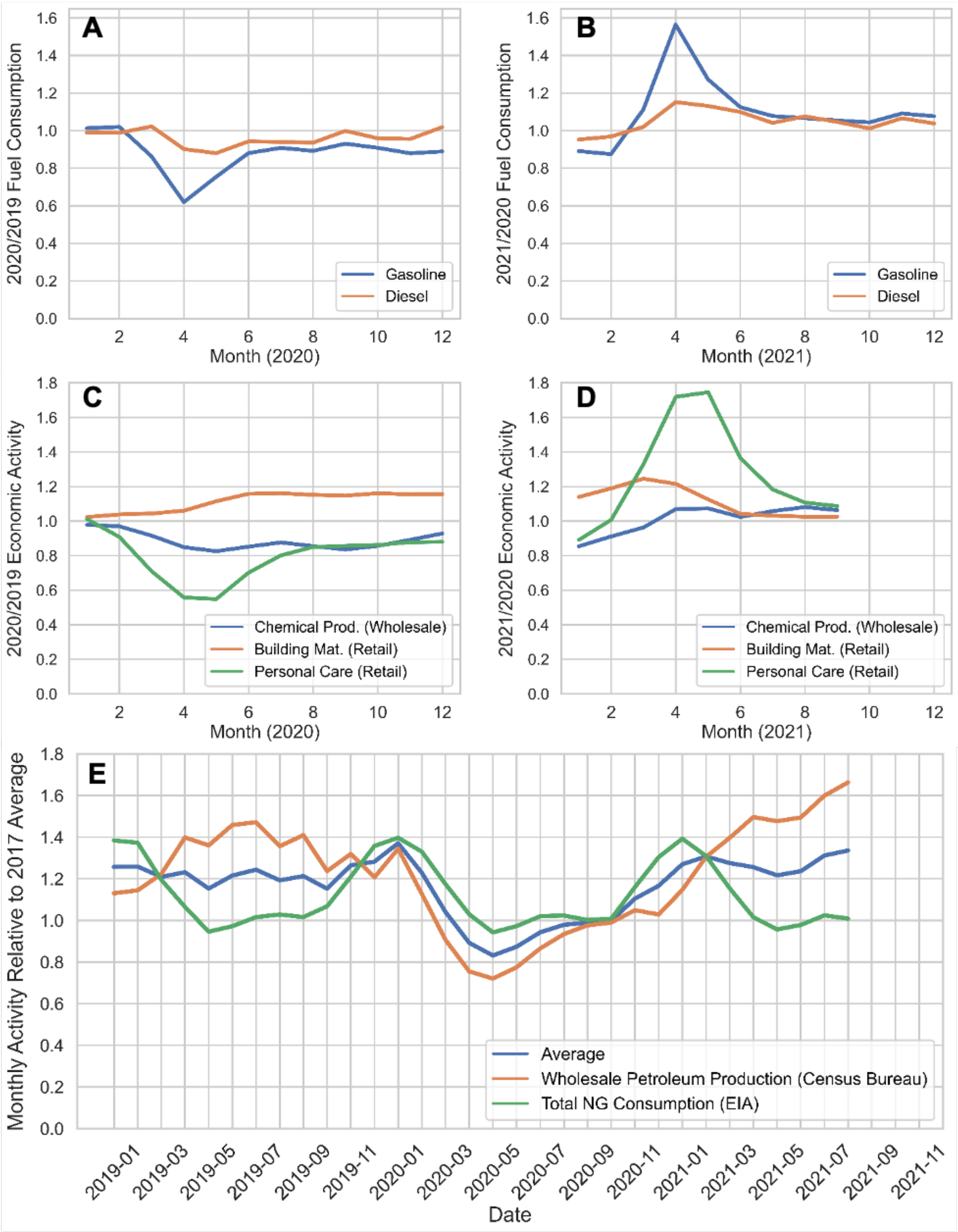

Fig. S1. Monthly emission adjustments for mobile sources, VCPs, and oil & gas. A-B: 2020 and 2021 fuel consumption used in mobile source emissions adjustments; C-D: 2020 and 2021 economic activity used in VCP emissions adjustments; E: activity in petroleum production and natural gas consumption from 2019-2021.

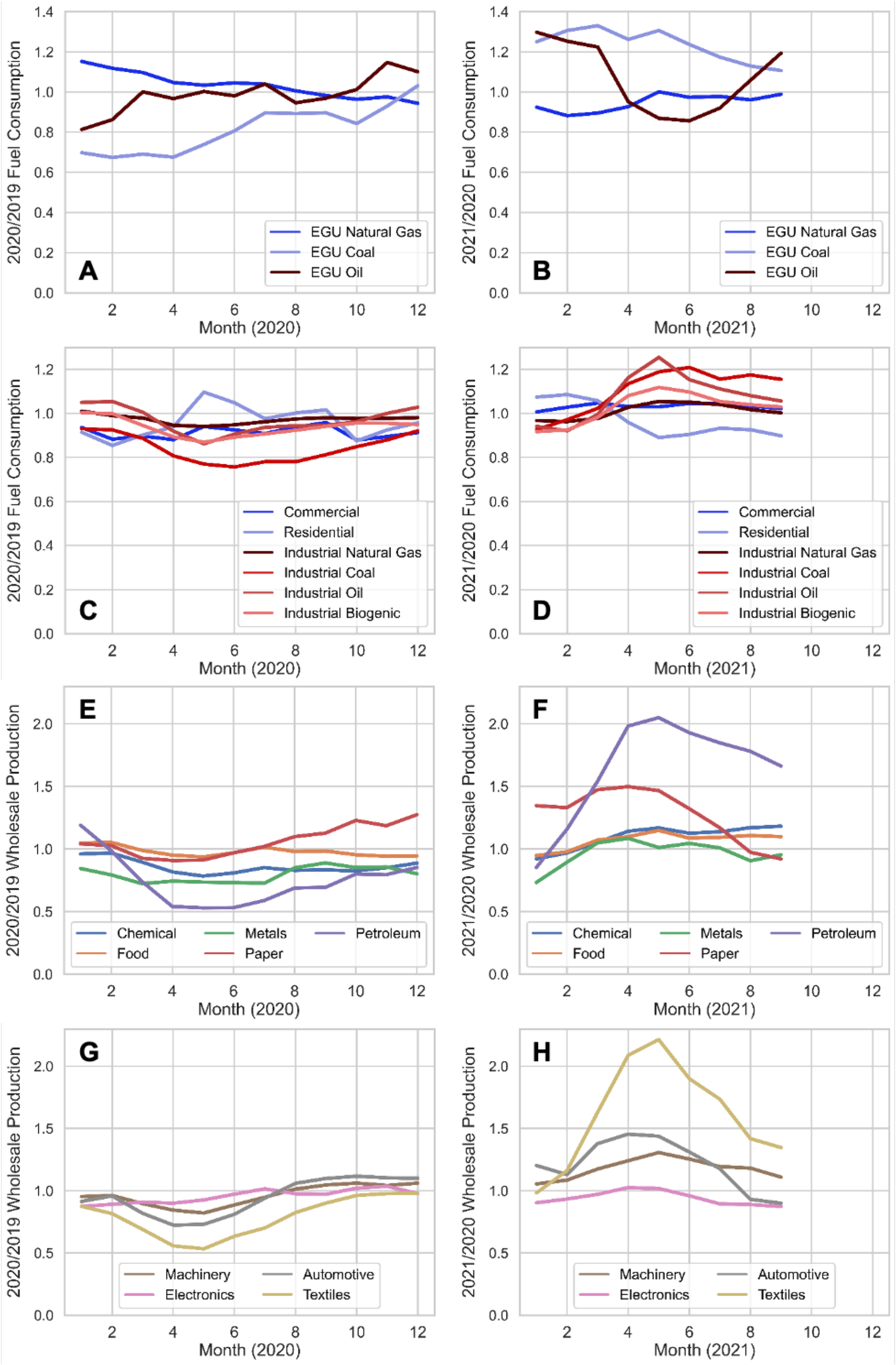

Fig. S2. Monthly emission adjustments for other anthropogenic sectors. A-B: 2020 and 2021 fuel consumption by electricity generation units; C-D: 2020 and 2021 fuel consumption by the industrial, residential and commercial Sectors; E-H: wholesale production activity in 2020 and 2021.

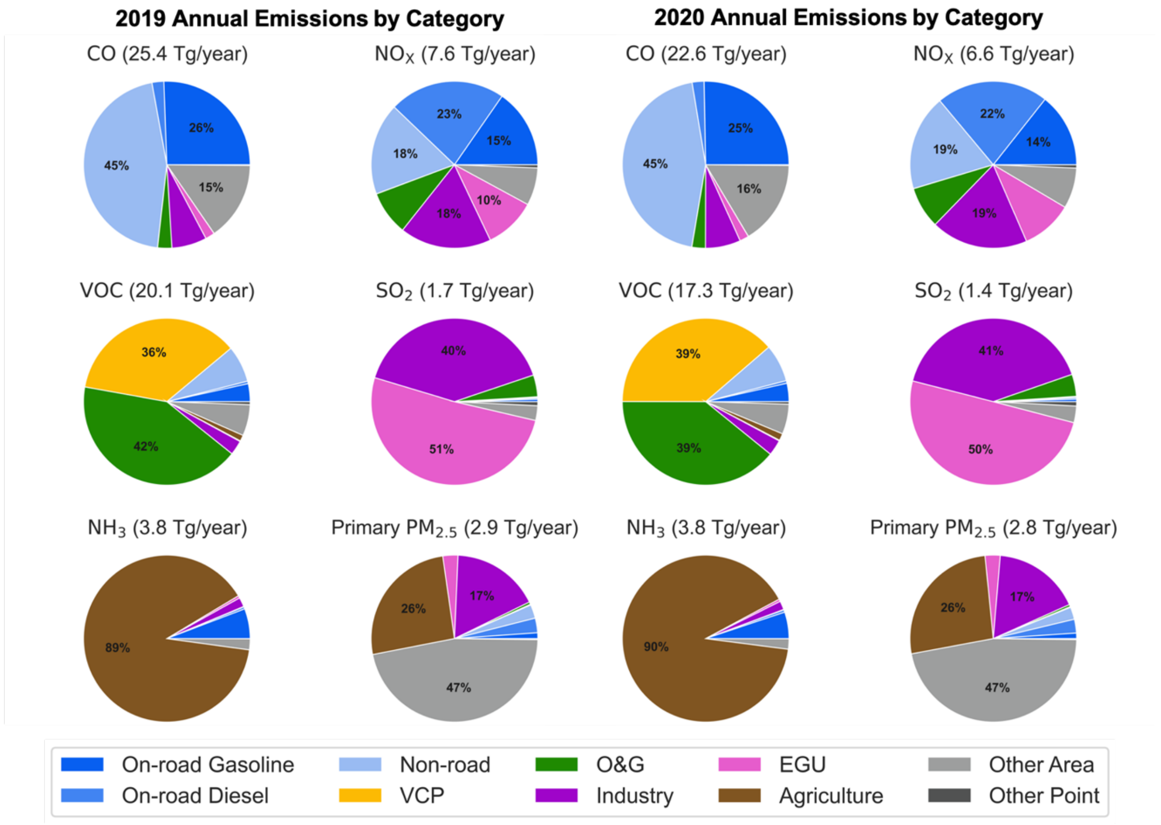

Fig. S3. Fractional contributions of source sectors for 2019 (left panel) and 2020 (right panel). Annual total emissions for each species are shown above each pie chart.

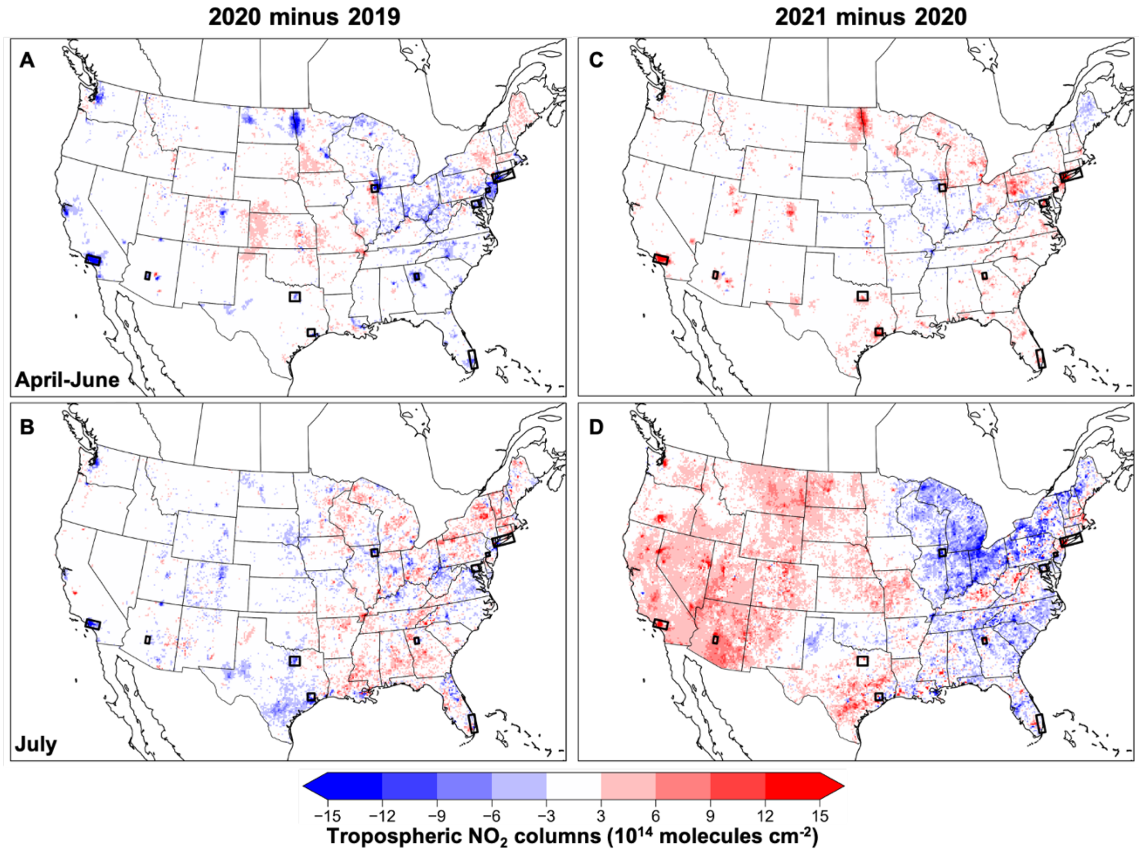

Fig. S4. TROPOMI NO_2 column changes between 2019 and 2020 (A&B), and between 2020 and 2021 (C&D) for April-June (A&C) and July (B&D). Black boxes represent top 10 populated cities. Much higher NO_2 columns over western US in panel D are mainly due to fires.

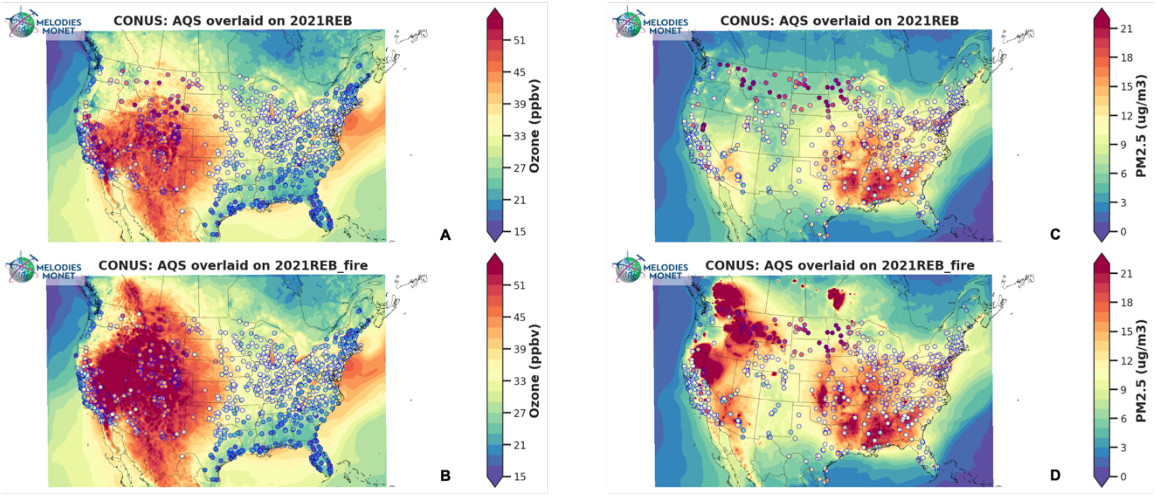

Fig. S5. Comparisons of simulated surface ozone (A&B) and fine particles (C&D) against AQS observations for July 2021. A&C: simulations without fire emissions; B&D: simulations with fire emissions. Circles overlaid on model simulated air pollutants represent observed concentrations from AQS surface monitoring sites.

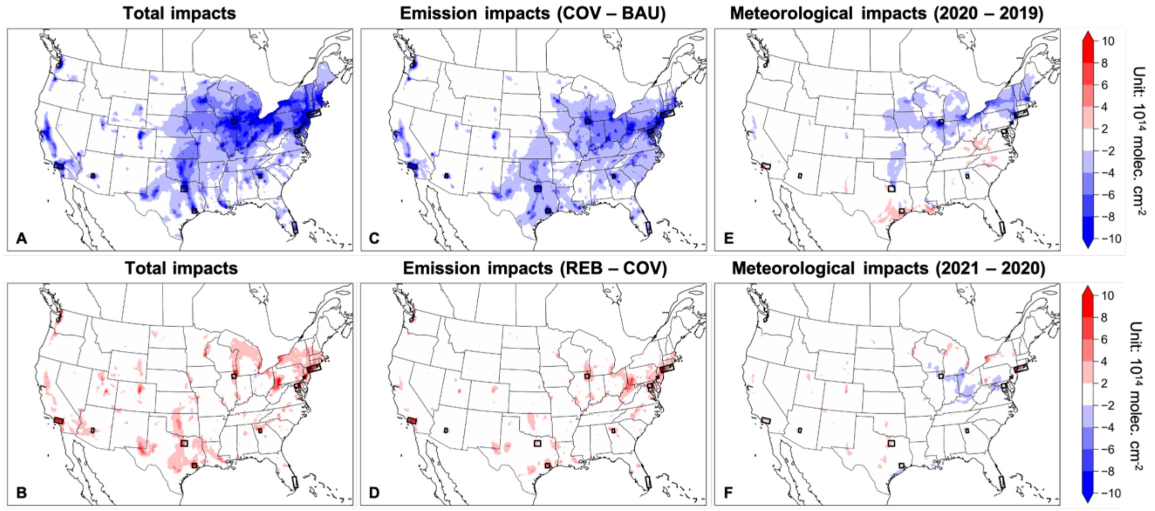

Fig. S6. Comparison of simulated NO_2 columns for April-June 2019, 2020, and 2021 over the US. A&B: difference in NO_2 columns between 2019 and 2020 (A) and between 2020 and 2021 (B); C&D: difference in NO_2 columns due to emission changes from business-as-usual condition (BAU) to COVID condition (COV, C), and from COV to rebounded emission condition (REB, D); E&F: difference in NO_2 columns due to meteorological changes from 2019 condition to 2020 condition (E) and from 2020 condition to 2021 condition (F). Black boxes indicate top 10 populated cities.

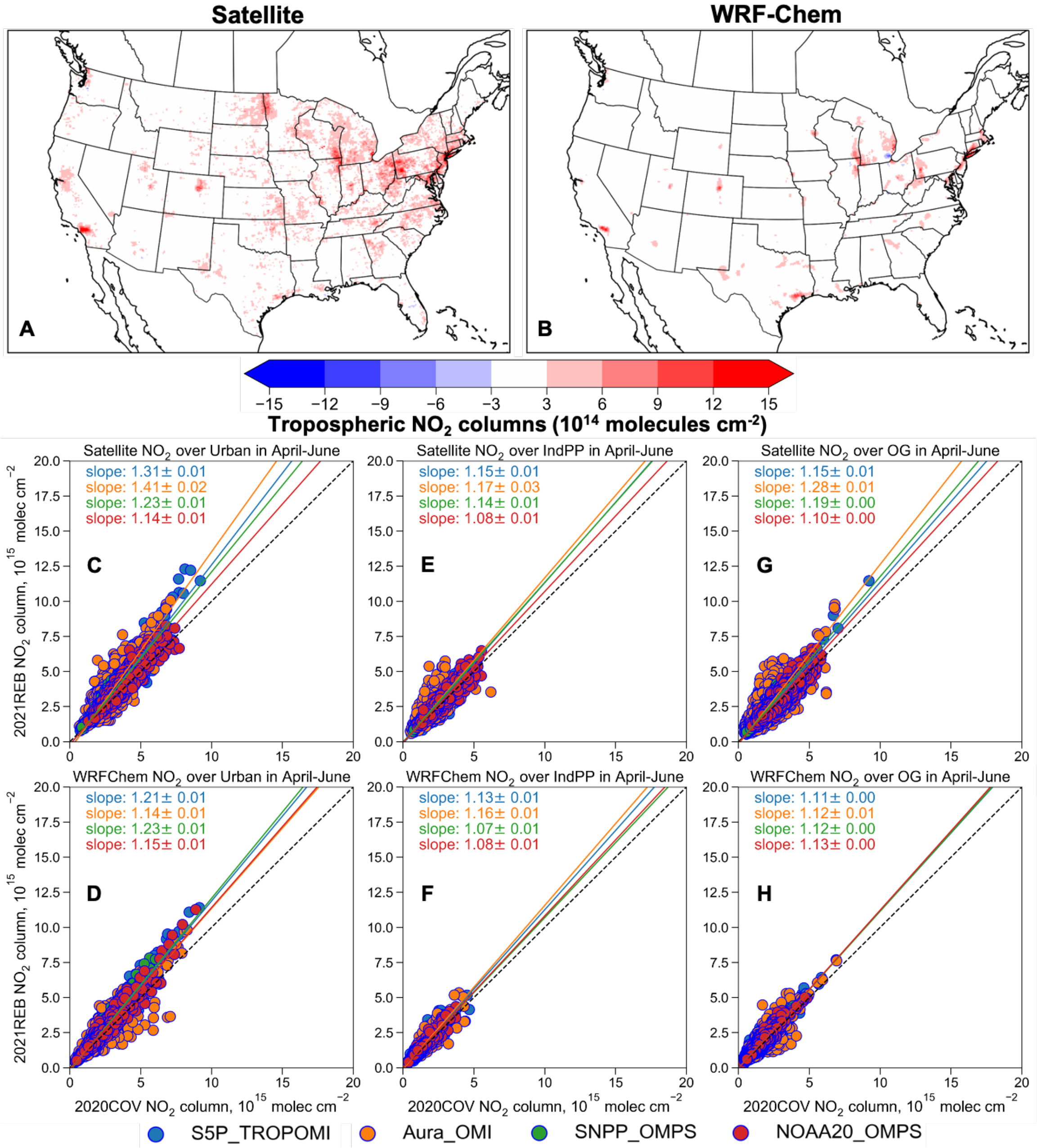

Fig. S7. Evaluation of simulated tropospheric NO_2 column concentrations with multiple satellite observations during April-June between 2020 and 2021. A: Observed NO_2 changes based on the average of four satellite data (S5P TROPOMI, Aura OMI, S-NPP OMPS, and NOAA-20 OMPS) with air mass factors or shape factors in the satellite products replaced by the model profiles; B: Model simulated NO_2 changes based on the average of resampled model data along each satellite track; C-H: NO_2 columns over urban (C&D), industrial/power plant (E&F), and oil & gas (G&H) source regions from satellite data (C, E, and G) and model estimates (D, F, and H) for 2020 COVID scenario (2020COV, x axis) and 2021 rebounded emission scenario (2021REB, y axis). Slope is calculated based on the orthogonal distance regression with 95% confidence interval.

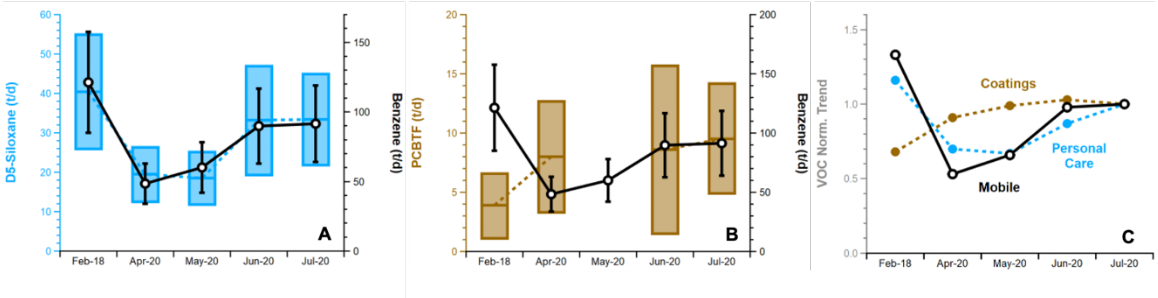

Fig. S8. Evaluation of VCP emissions with ground-based measurement at Boulder. A: ambient derived emission changes for D5-Siloxane from personal care product (left Y axis, blue bars) and Benzene from mobile sources (right Y axis, solid black lines). B: ambient derived emission changes for PCBTF from solvent-based coatings (left Y axis, tan bars) with emission changes of Benzene from mobile sources (solid black lines) shown on the right Y axis; C: Change in retail sales of Building Material stores (proxy for coatings, brown line) and Health and Personal Care stores (blue line) (https://www.census.gov/retail/index.html).

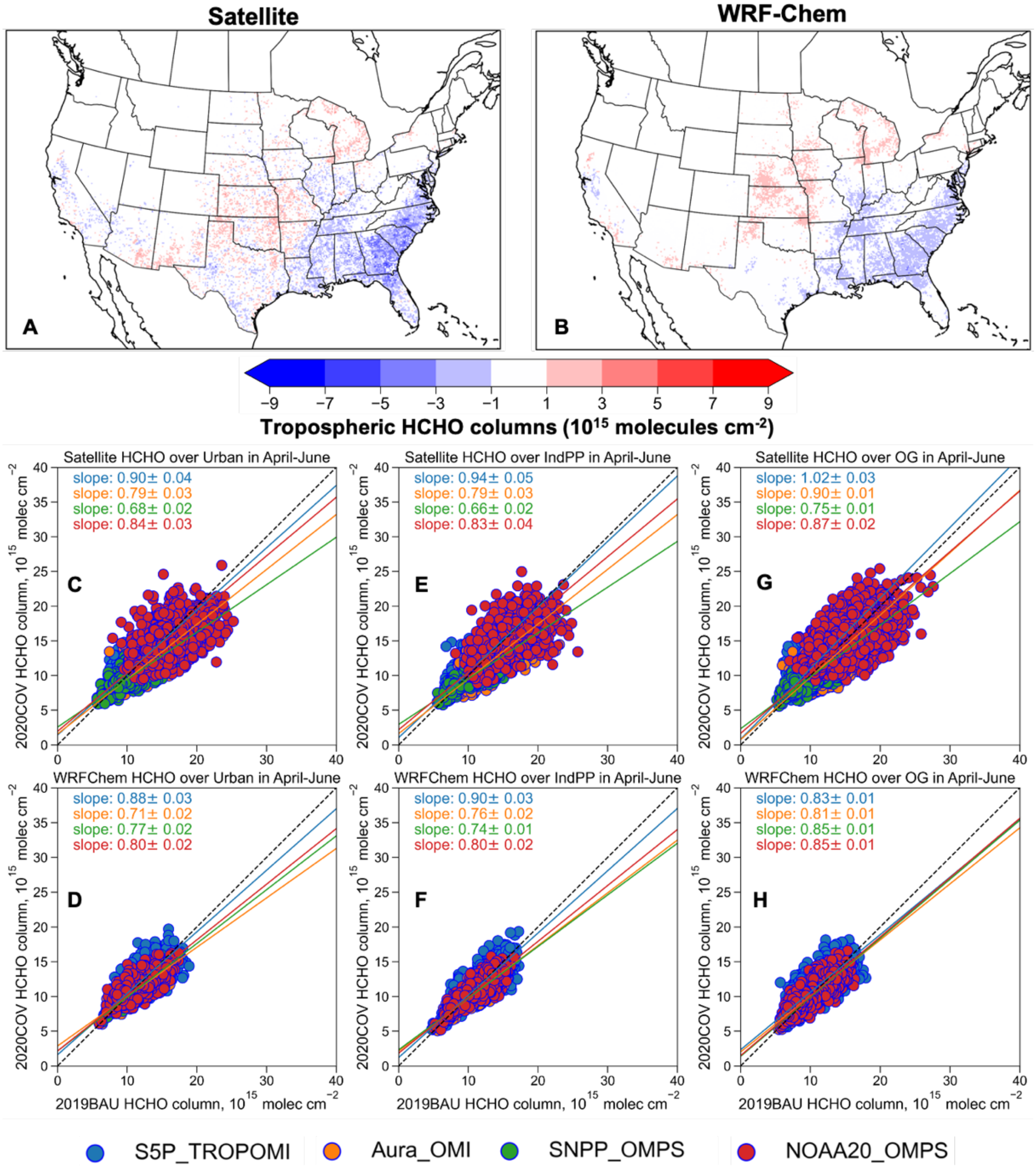

Fig. S9. Evaluation of simulated tropospheric HCHO column concentrations with multiple satellite observations during April-June between 2019 and 2020. A: Observed HCHO changes based on the average of four satellite data (S5P TROPOMI, Aura OMI, S-NPP OMPS, and NOAA-20 OMPS) with air mass factors or shape factors in the satellite products replaced by the model profiles; B: Model simulated HCHO changes based on the average of resampled model data along each satellite track; C-H: HCHO columns over urban (C&D), industrial/power plant (E&F), and oil & gas (G&H) source regions from satellite data (C, E, and G) and model estimates (D, F, and H) for 2019 business-as-usual scenario (2019BAU, x axis) and 2020 COVID scenario (2020COV, y axis). Slope is calculated based on the orthogonal distance regression with 95% confidence interval.

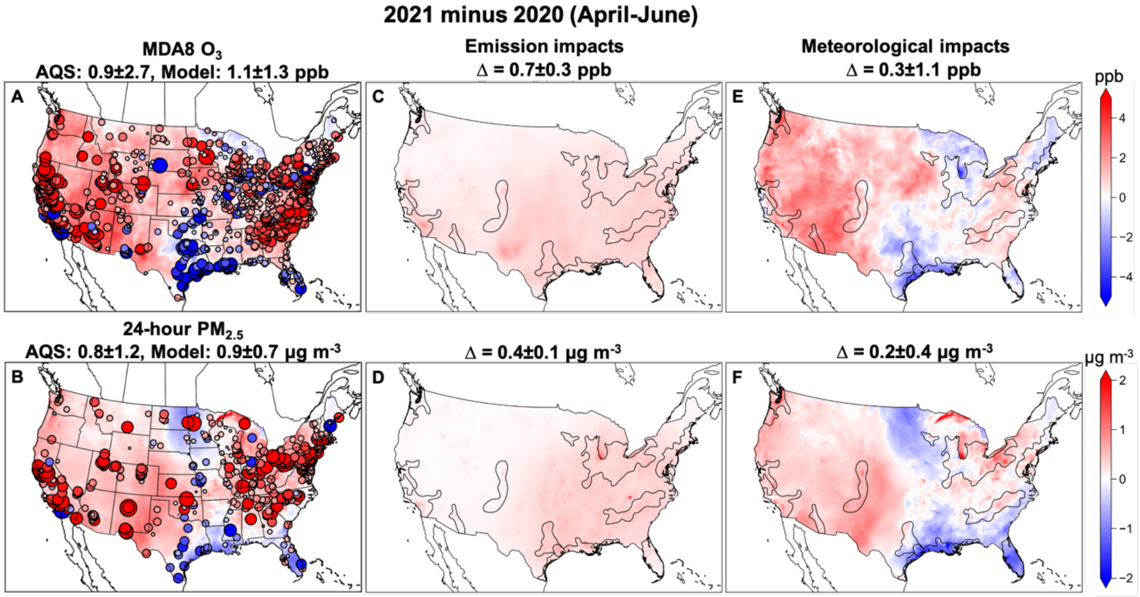

Fig. S10. April-June changes in MDA8 O_3 (upper panel, A, C, and E) and 24-hour averaged $PM_{2.5}$ (lower panel, B, D, and F) from 2020 to 2021. A&B: circles overlaid on model simulated air quality changes represent observed changes from AQS surface monitoring sites with size in proportion to the absolute changes; site-averaged changes ± standard deviation from AQS observations and model estimates are shown above each figure. C-F: air quality impacts due to emission changes only (C&D) and due to meteorological variability only (E&F); groups of metropolitan areas are shown in black polylines; population-weighted averaged changes ± standard deviation from model grids are shown above on each figure.

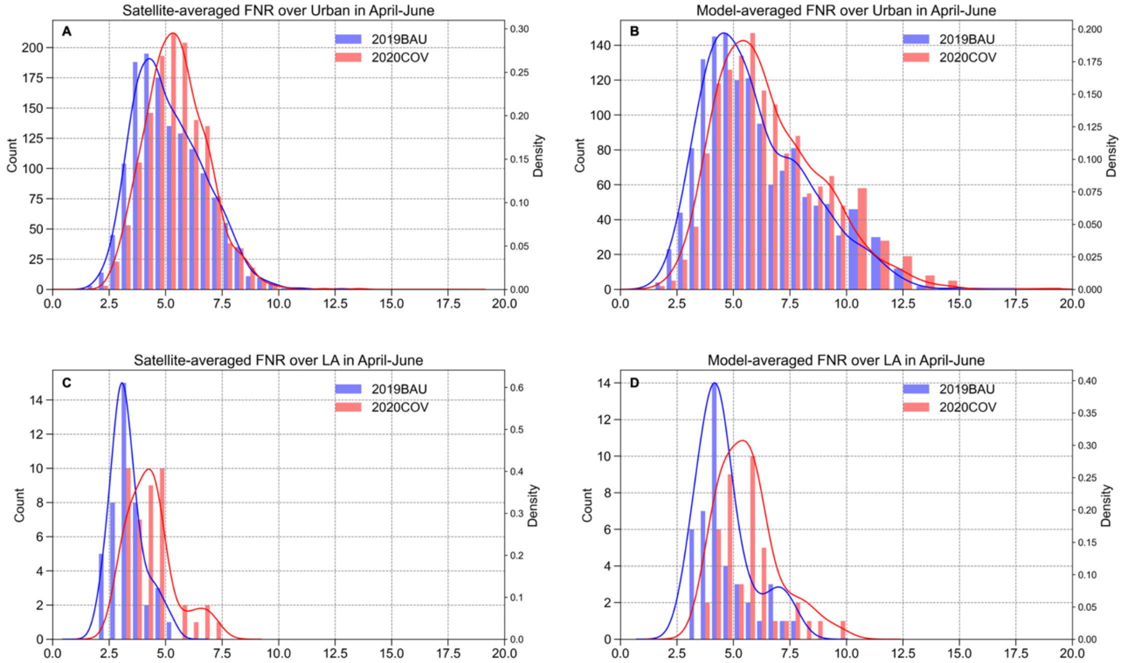

Fig. S11. Evaluation of simulated tropospheric column HCHO/NO_2 ratios (FNR) with multiple satellite observations during April-June under 2019 business-as-usual scenario (2019BAU) and 2020 COVID scenario (2020COV). A&C: Observed FNR based on the average of four satellite data (S5P TROPOMI, Aura OMI, S-NPP OMPS, and NOAA-20 OMPS); B&D: Model simulated FNR based on the average of resampled model data along each satellite track; A&B: FNR over urban source regions; C&D: FNR over Los Angeles; Urban transitional regime with FNR in the range of 3.0 to 4.5 and in the range of 4.1 to 5.0 over Los Angeles based on Jin et al. (30). The count in the histogram figures (Y axis) represents the number of urban grids that fall into each FNR bin and the density (X axis) represents the probability density.

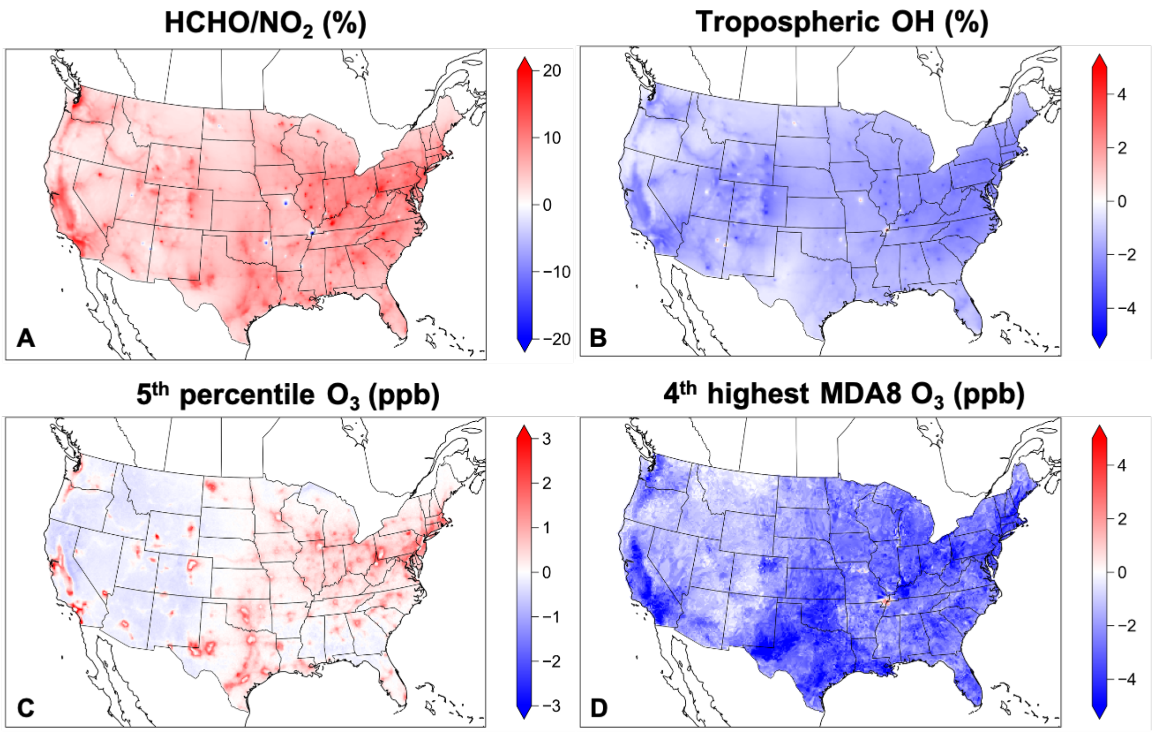

Fig. S12. Emission impacts on $HCHO/NO_2$ ratio (A, percentage change %), tropospheric OH concentrations (B), 5th percentile hourly O_3 (C) and 4th highest MDA8 O_3 (D) during April 2020 to March 2021.

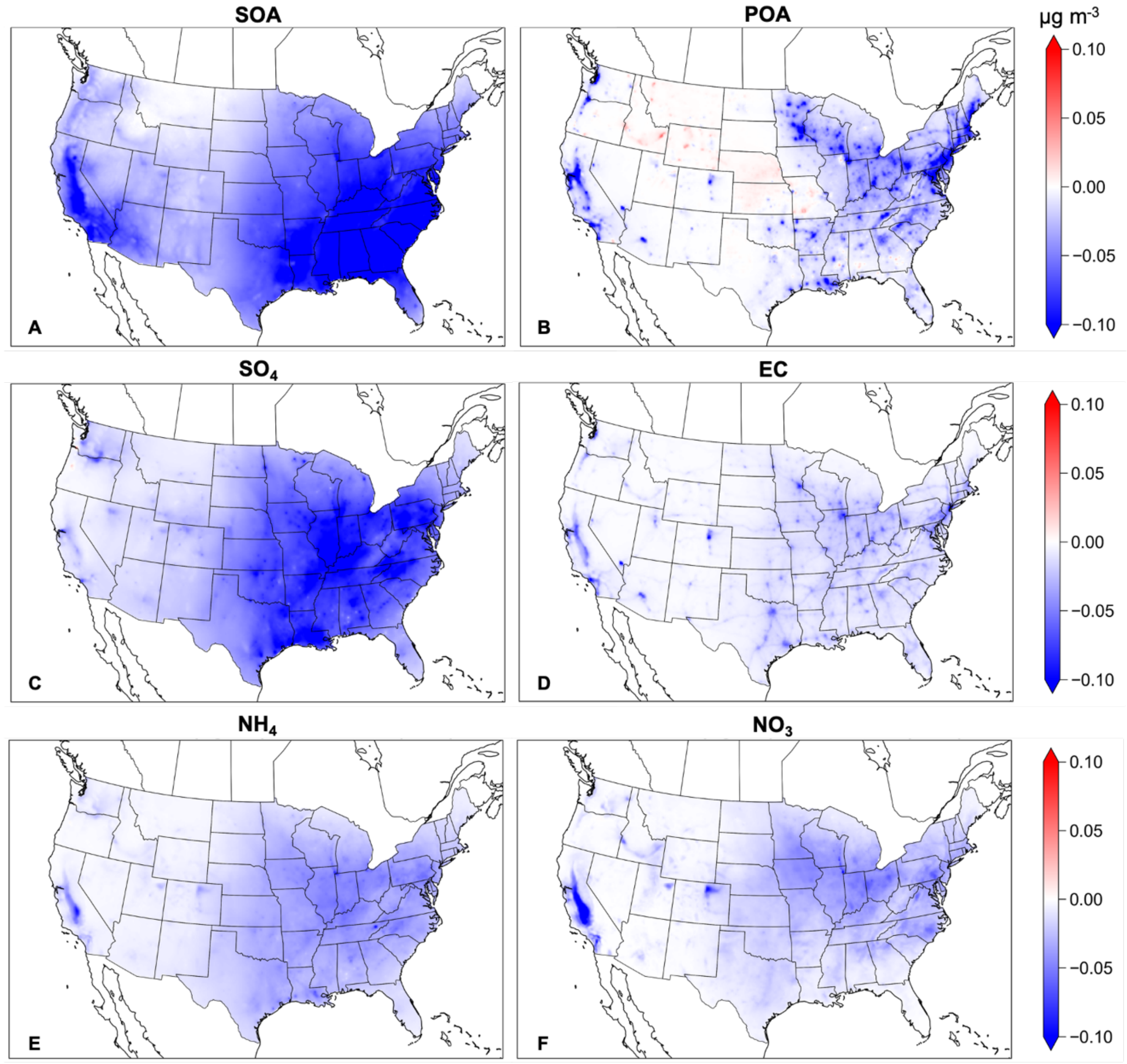

Fig. S13. Impacts on $PM_{2.5}$ components (A: secondary organic aerosol, SOA; B: primary organic aerosol, POA; C: sulfate, SO_4; D: element carbon, EC; E: ammonium, NH_4; F: nitrate, NO_3) due to COVID-induced emission changes during April 2020 to March 2021.

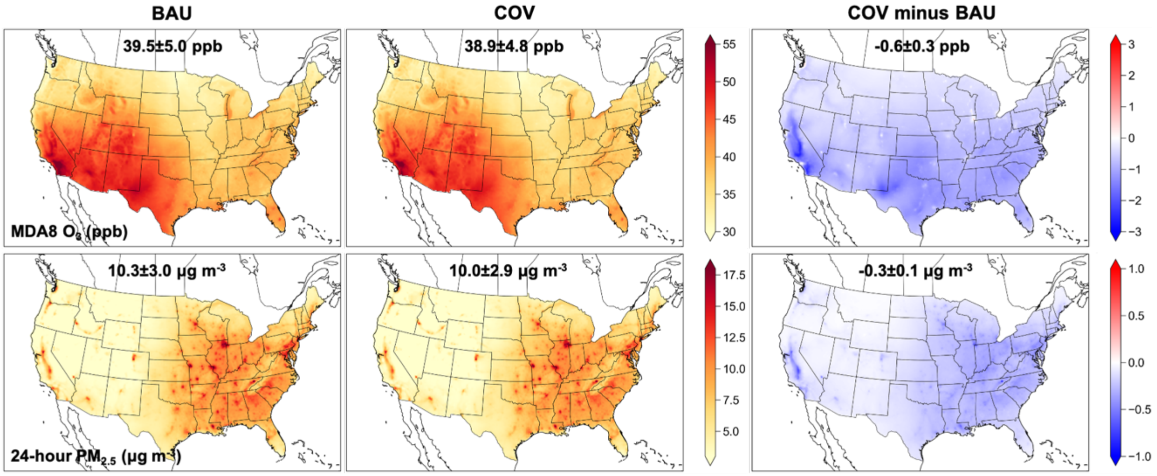

Fig. S14. Comparison of annual mean MDA8 O_3 (upper panel) and 24-hour averaged $PM_{2.5}$ (lower panel) for the period of April 2020 to March 2021 between business-as-usual emission condition (BAU) and COVID emission condition (COV). Population weighted averages with standard deviation based on all grid cells are shown on each figure.

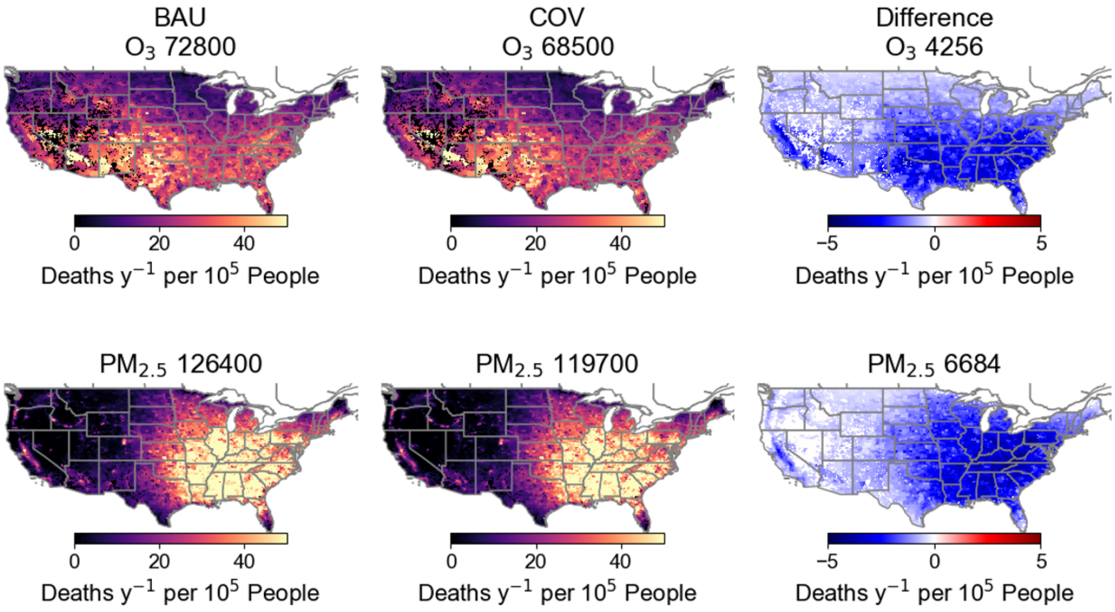

Fig.15. O_3 and $PM_{2.5}$ attributable deaths (per year per 10^5 people) based on business-as-usual scenario (BAU, A&D) and COVID scenario (COV, B&E) for the period of April 2019 to March 2020. The total attributable deaths due to each air pollutant and scenario are shown above each figure. C&F: difference in attributable deaths between BAU and COV scenarios. The difference in the total attributable deaths are shown above each figure.

Table S1. Summary of Anthropogenic Emission Sectors and COVID-19 Pandemic Scaling Factor Data.

Sector	Base Inventory	SCC Codes	COVID Scaling Data	Spatial Adjustment	Ref.
**On-Road**					
Gasoline	FIVE18	2200000000-2209999999	EIA Gasoline[a]	State	McDonald et al. (4)
Diesel	FIVE18	2230000000-2239999999	EIA Diesel[b]	State & US	McDonald et al. (4)
**Non-Road**					
Agricultural Diesel	FIVE18	2270005000-2270005999	EIA Diesel[b]	PADD	McDonald et al. (4)
Non-Ag Diesel	FIVE18	2270000000-2270004999<br>2270006000-2270099999	EIA Diesel[b]	PADD	McDonald et al. (4)
Gasoline (Marine)	FIVE18	2282005000-2282005025<br>2282010000-2282010025	EIA Gasoline[a]	PADD	McDonald et al. (4)
Gasoline (2-Stroke)	FIVE18	2260000000-2260010010	EIA Gasoline[a]	PADD	McDonald et al. (4)
Gasoline (4-Stroke)	FIVE18	2265000000-2265010010	EIA Gasoline[a]	PADD	McDonald et al. (4)
**Point**					
Electricity Generation					
Coal	NEI17	10100101-10100318<br>10100801-10100801<br>20100301-20100301	CEMS & EIA EGU Coal[d]	Facility & US	EPA (1)
Oil	NEI17	10100401-10100505<br>20100101-20100109<br>20100901-20100909	CEMS & EIA EGU Oil[d]	Facility & US	EPA (1)
Natural Gas	NEI17	10100601-10100712<br>10101001-10101002<br>20100201-20100209<br>20100702-20100707	CEMS & EIA EGU NG[d]	Facility & US	EPA (1)

Sector	Base Inventory	SCC Codes	COVID Scaling Data	Spatial Adjustment	Ref.
Biomass	NEI17	10100901-10100912<br>10101101-10102101<br>20100801-20100809	CEMS & EIA EGU Biomass[d]	Facility & US	EPA (1)
Industrial Boilers					
Coal	NEI17	10200101-10200307<br>10200802-10200804<br>39000199-39000399<br>39000801-39000899	EIA Industrial Coal[e]	US	Xing et al. (25)
Oil	NEI17	10200401-10200506<br>10500105<br>10500113-10500114<br>20200101-20200109<br>20200401-20200506<br>20200901-20200909<br>20201701-20201707<br>20400101-20400499<br>39000402-39000599<br>39900501<br>39901001	EIA Industrial Oil[e]	US	Xing et al. (25)
Natural Gas	NEI17	10200601-10200799<br>10201001-10201003<br>10500106-10500110<br>20200201-20200256<br>20200702-20200714<br>20201001-20201013<br>39000602-39000797<br>39001099<br>39900601-39900701	EIA Industrial NG[e]	US	Xing et al. (25)
Biomass	NEI17	10200901-10200912<br>10201101-10201902<br>20201602-20201609<br>39000989-39000999<br>39001289-39001399<br>39900721-39900801<br>39901601-39901601	EIA Industrial Biomass[e]	US	Xing et al. (25)
Commercial Boilers					

Sector	Base Inventory	SCC Codes	COVID Scaling Data	Spatial Adjustment	Ref.
Coal	NEI17	10300101-10300306	EIA Commercial[f]	US	Xing et al. (25)
Oil	NEI17	10300401-10300505<br>10500205<br>10500213-10500214<br>20300101-20300109<br>20300301-20300401<br>20300901	EIA Commercial[f]	US	Xing et al. (25)
Natural Gas	NEI17	10300601-10300799<br>10301001-10301003<br>10500206<br>10500210<br>20300201-20300209<br>20301001-20301007	EIA Commercial[f]	US	Xing et al. (25)
Biomass	NEI17	10300811-10300912<br>10301101-10301303<br>10500209<br>20300701-20300809	EIA Commercial[f]	US	Xing et al. (25)
Industrial Processes					
Chemical	NEI17	30100101-30199998<br>30800101-30899999<br>64470010-68510001	DOC Chemicals [NAICS #4246][g]	US	--
Food	NEI17	30200101-30299998<br>62540023-62540024	DOC Grocery [NAICS #4244][g]	US	--
Metals & Mining	NEI17	30300001-30599999<br>30900198-30999999	DOC Metals [NAICS #4235][g]	US	--
Refinery & Bulk Terminals	NEI17	30600102-30699999<br>31700101-31700101<br>39090001-39092056<br>40301001-40400279<br>40400401-40400498<br>40600101-40600299<br>40600301-40688801<br>40700401-49099998	DOC Petroleum [NAICS #4247][g]	US	--
Pulp, Paper & Construction	NEI17	30700101-30799999<br>31100102-31100299	DOC Lumber & Const. [NAICS #4233][g]	US	--

Sector	Base Inventory	SCC Codes	COVID Scaling Data	Spatial Adjustment	Ref.
Electrical Equipment	NEI17	31300500-31399999	DOC Electronics [NAICS #4236][g]	US	--
Automotive	NEI17	31400901-31499999	DOC Motor Vehicle [NAICS #4231][g]	US	--
Apparel	NEI17	32099997-33088801	DOC Apparel [NAICS #4243][g]	US	--
Photographic Film	NEI17	31501001-31501002<br>31603001-31616006	DOC Professional Supplies [NAICS #4234][g]	US	--
Drug Mfg.	NEI17	31502001-31503102	DOC Drug [NAICS #4242][g]	US	--
Misc. Industry	NEI17	20101020-20190099<br>20280001-20282599<br>20380001-20380001<br>28888801-28888801<br>31504001-31505002<br>31801001-31801030<br>38500101-38500110<br>39902001-39999999	DOC Misc. Goods [NAICS #4249][g]	US	--
Oil & Gas Prod.	NEI17	31000101-31088811<br>40400300-40400340	DOC Petroleum [NAICS #4247][g]	US	
Point VCPs					
Ind. Degreasing	VCP18	40100101-40188898	DOC Chemicals [NAICS #4246][g]	US	[5]
Ind. Coatings	VCP18	40200101-40200601<br>40200801-40299998	DOC Chemicals [NAICS #4246][g]	US	[5]
Ind. Adhesives	VCP18	40200701-40200712	DOC Chemicals [NAICS #4246][g]	US	[5]
Printing Inks	VCP18	36000101-36000103<br>40500204-40500806	DOC Chemicals [NAICS #4246][g]	US	[5]
Airports	NEI17	2265008005-2275001000	BTS Air Carrier Traffic[h]	US	--
Railyard Equipment	NEI17	28500201-28500201	BTS Rail[c]	US	--
**Area**					
Industrial Boilers					

Sector	Base Inventory	SCC Codes	COVID Scaling Data	Spatial Adjustment	Ref.
Coal	NEI17	2102001000-2102002000	EIA Industrial Coal[e]	US	Xing et al. (25)
Oil	NEI17	2102004000-2102005000 2102011000	EIA Industrial Oil[e]	US	Xing et al. (25)
Natural Gas	NEI17	2102006000-2102007000 2102010000	EIA Industrial NG[e]	US	Xing et al. (25)
Biomass	NEI17	2102008000	EIA Industrial Biomass[e]	US	Xing et al. (25)
Commercial Boilers					
Coal	NEI17	2103001000-2103002000	EIA Commercial[f]	US	Xing et al. (25)
Oil	NEI17	2103004000-2103005000 2103011000	EIA Commercial[f]	US	Xing et al. (25)
Natural Gas	NEI17	2103006000-2103007000	EIA Commercial[f]	US	Xing et al. (25)
Biomass	NEI17	2103008000	EIA Commercial[f]	US	Xing et al. (25)
Residential Boilers					
Coal	NEI17	2104001000-2104002000	EIA Residential[i]	US	Xing et al. (25)
Oil	NEI17	2104004000 2104011000	EIA Residential[i]	US	Xing et al. (25)
Natural Gas	NEI17	2104006000-2104007000	EIA Residential[i]	US	Xing et al. (25)
Biomass	NEI17	2104008100-2104009000	EIA Residential[i]	US	Xing et al. (25)
Industrial Processes					
Chemical	NEI17	2301000000-2301030000 2308000000	DOC Chemicals [NAICS #4246][g]	US	--
Food	NEI17	2302000000-2302080002	DOC Grocery [NAICS #4244][g]	US	--
Metals & Mining	NEI17	2304000000-2305000000	DOC Metals [NAICS #4235][g]	US	--

Sector	Base Inventory	SCC Codes	COVID Scaling Data	Spatial Adjustment	Ref.
		2309000000<br>2325000000-<br>2325060000			
Pulp, Paper & Construction	NEI17	2307000000<br>2311010000-<br>2311030000	DOC Lumber & Const. [NAICS #4233][g]	US	--
Machinery	NEI17	2312000000	DOC Machinery [NAICS #4238][g]	US	--
Misc. Industry	NEI17	2306010000-<br>2306010100<br>2399000000-<br>2399010000	DOC Misc. Goods [NAICS #4249] [g]	US	--
Oil & Gas (onshore)	FOG18 & NEI17	2310000220-<br>2310001000<br>2310010100-<br>2310011600<br>2310020000-<br>2310021803<br>2310023000-<br>2310111701<br>2310121100-<br>2310121700<br>2310300220-<br>2310421603	DrillingInfo[l] for FOG emissions<br><br>Average scaling of DOC Petroleum [NAICS #4247][g] & EIA Total Natural Gas Consumption[d,e,f,i] for NEI17 emissions	US	Francoeur et al. (24)
Oil & Gas (offshore)	NEI17	2310002000-<br>2310002421<br>2310012000-<br>2310012526<br>2310022000-<br>2310022506<br>2310112401<br>2310122100	DOC Petroleum [NAICS #4247][g]	US	--
Storage & Transport	NEI17	2505010000-<br>2525000000	DOC Petroleum [NAICS #4247][g]	US	--
Agriculture					

Sector	Base Inventory	SCC Codes	COVID Scaling Data	Spatial Adjustment	Ref.
Crop	NEI17	2801000000-2801000008 2801520000-2801700099	Unscaled	--	--
Burning	NEI17	2801500000-2801500600	Unscaled	--	--
Livestock	NEI17	2805001000-2807030000	Unscaled	--	--
Area VCPs					
Architectural Coatings	VCP18	2401001000 2460400000-2460500000	Retail Building Materials [NAICS #4441][k]	US	McDonald et al. (9)
Ind. Coatings	VCP18	2401005000-2401200000	DOC Chemicals [NAICS #4246][g]	US	McDonald et al. (9)
Ind. Degreasing	VCP18	2415000000	DOC Chemicals [NAICS #4246][g]	US	McDonald et al. (9)
Printing Inks	VCP18	2425000000-2425040000	DOC Chemicals [NAICS #4246][g]	US	McDonald et al. (9)
Ind. Adhesives	VCP18	2440020000	DOC Chemicals [NAICS #4246][g]	US	McDonald et al. (9)
Personal Care	VCP18	2460100000	Retail Health & Personal Care [NAICS #446] less Retail Pharmacy & Drug Stores [NAICS #44611][k]	US	McDonald et al. (9)
Cleaning	VCP18	2420000000-2420000999 2460200000	Retail Health & Personal Care [NAICS #446] less Retail Pharmacy & Drug Stores [NAICS #44611][k]	US	McDonald et al. (9)
Consumer Adhesives	VCP18	2460600000	Retail Building Materials [NAICS #4441][k]	US	McDonald et al. (9)
Consumer Pesticides	VCP18	2460800000	Unscaled	US	McDonald et al. (9)
Agricultural Pesticides	VCP18	2461800001-2461850000	Unscaled	US	McDonald et al. (9)

Sector	Base Inventory	SCC Codes	COVID Scaling Data	Spatial Adjustment	Ref.
Comm. Marine Diesel	NEI17	2280002101-2280002204	DOC Shipping Weight[j]	US	--
Comm. Marine Res.	NEI17	2280003103-2280003204	DOC Shipping Weight[j]	US	--
Locomotives	NEI17	2285002006-2285002010	BTS Rail[c]	US	--
Waste	NEI17	2601000000-2680003000	Unscaled	US	--
Dust	NEI17	2294000000-2296000000	Unscaled	US	--
Miscellaneous	NEI17	2810003000-2862000000	DOC Misc. Goods [NAICS #4249] [g]	US	--

a. Monthly gasoline sales data can be found from the US Energy Information Administration at: https://www.eia.gov/dnav/pet/pet_cons_prim_a_EPM0_P00_Mgalpd_m.htm

b. Monthly diesel sales data can be found from the US Energy Information Administration at: https://www.eia.gov/dnav/pet/pet_cons_prim_a_EPD2_P00_Mgalpd_m.htm

c. Monthly Carloads + Intermodal Units from the US Bureau of Transportation Statistics: https://data.bts.gov/stories/s/m9eb-yevh

d. Continuous emissions monitoring data is only used for NO_x and SO_2. Other pollutants are scaled using EIA data. Continuous emissions monitoring data can be found at: https://campd.epa.gov/data . Monthly fuel consumption for the electricity power sector can be found from the US Energy Information Administration in Table 2.6 at: https://www.eia.gov/totalenergy/data/monthly/index.php

e. Monthly fuel consumption by the industrial sector can be found from the US Energy Information Administration in Table 2.4 at: https://www.eia.gov/totalenergy/data/monthly/index.php

f. All commercial fuel scalings use the total fuel consumption (Coal + NG + Oil + Biomass) from the commercial sector to calculate the scaling factor. Monthly fuel consumption by the commercial sector can be found from the US Energy Information Administration in Table 2.3 at: https://www.eia.gov/totalenergy/data/monthly/index.php

g. Monthly wholesale trade data from the US Census Bureau can be found at: https://www.census.gov/wholesale/index.html

h. Monthly Revenue Miles Flown (Passenger + Cargo) from the US Bureau of Transportation Statistics at: https://www.transtats.bts.gov/TRAFFIC/

i. All residential fuel scalings use the total fuel consumption (Coal + NG + Oil + Biomass) from the residential sector to calculate the scaling factor. Monthly fuel consumption by the residential sector can be found from the US Energy Information Administration in Table 2.2 at: https://www.eia.gov/totalenergy/data/monthly/index.php

j. Monthly Shipping Weight (Imports+Exports) from the US Census Bureau can be found at: https://www.census.gov/data/developers/data-sets/international-trade.html

k. Monthly Retail Sales from the US Census Bureau can be found at: https://www.census.gov/retail/index.html

l. Well-level production and drilling data from Enverus DrillingInfo database.

Table S2. NOAA CSL WRF-Chem Model Configuration[a]

**Settings**	**Description**
Horizontal Resolution	12 km x 12 km
Vertical Resolution	50 levels (up to 50 hPa)
Meteorology	North American Mesoscale Model
Surface Layer	Mellor-Yamada Nakanishi and Niino
Planetary Boundary Layer	Mellor-Yamada Nakanishi and Niino Level 2.5
Cumulus Scheme	Grell-Devenyi (GD) Ensemble Cumulus
Land Surface	Noah Land Surface Model
Microphysics	WRF Single Moment 5-Class
Short- and Long-Wave Radiation	Rapid Radiative Transfer Model for General Circulation Models
Gas-Phase Chemistry	RACM-ESRL-VCP (updated oxy-VCP chemistry)
Photolysis	Madronich Photolysis (TUV)

a. See https://www2.mmm.ucar.edu/wrf/users/docs/user_guide_v4/contents.html for full description of model options.

Table S3. Summary of WRF-Chem simulations conducted in this work

Experiments	Description
2019BAU	Simulation driven by 2019 NAM meteorology and 2019 business as usual emission inventories (BAU) through April to June
2019BAU_fire	Simulation driven by 2019 NAM meteorology, 2019 business as usual emission inventories (BAU), and 2019 RAVE emissions for July only
2020BAU	Simulation driven by 2020 NAM meteorology and 2019 business as usual emission inventories (BAU) through April to June
2020COV	Simulation driven by 2020 NAM meteorology and 2020 COVID adjusted emission inventories (COV) through April to June
2020COV_fire	Simulation driven by 2020 NAM meteorology, 2020 COVID adjusted emission inventories (COV), and 2020 RAVE emissions for July only
2021COV	Simulation driven by 2021 NAM meteorology and 2020 COVID adjusted emission inventories (COV) through April to June
2021REB	Simulation driven by 2021 NAM meteorology and 2021 rebounded emission inventories (REB) through April to June
2021REB_fire	Simulation driven by 2021 NAM meteorology, 2021 rebounded emission inventories (REB), and 2021 RAVE emissions for July only
BAU	Simulation driven by NAM meteorology and BAU emissions through April 2020 to March 2021
COV	Simulation driven by NAM meteorology and COV emissions through April 2020 to March 2021
BAU19	Simulation driven by NAM meteorology and BAU emissions through April 2019 to March 2020
COV19	Simulation driven by NAM meteorology and COV emissions through April 2019 to March 2020

Table S4. Statistics of model-AQS comparison (April-June 2019, 2020, and 2021) over the US[a]

	MDA8 O_3 (ppb)			24-hour $PM_{2.5}$ ($\mu g\ m^{-3}$)			T2 (K)		
	2019	2020	2021	2019	2020	2021	2019	2020	2021
Obs_Mean	45.84	44.79	45.86	6.95	6.91	7.77	290.13	290.68	291.29
Model_Mean	44.75	43.52	44.65	7.55	6.93	7.68	290.30	290.78	291.26
Mean_Bias	-1.09	-1.27	-1.21	0.60	0.01	-0.09	0.17	0.10	-0.04
Median_Bias	-1.54	-1.82	-1.79	0.46	0.06	-0.12	0.10	0.06	-0.07
Normalized_Mean_Bias_(%)	-2.38	-2.83	-2.63	8.61	0.18	-1.15	0.06	0.03	-0.01
Normalized_Median_Bias_(%)	-3.34	-4.07	-3.89	7.45	1.06	-1.77	0.04	0.02	-0.02
Coefficient_of_Determination_(R^2)	0.35	0.38	0.40	0.13	0.08	0.11	0.92	0.91	0.92
Root_Mean_Square_Error	9.04	8.64	9.26	4.77	4.82	4.99	2.42	2.62	2.54
Index_of_Agreement	0.76	0.78	0.79	0.60	0.54	0.59	0.98	0.98	0.98

a. Statistics are calculated through a python-based diagnostic package MELODIES MONET (https://github.com/NOAA-CSL/MELODIES-MONET).

Table S5. Statistics of model-AQS comparison (July 2019, 2020, and 2021) over the US[a]

	2019BAU	2019BAU_fire	2020COV	2020COV_fire	2021REB	2021REB_fire
O_3						
Mean_Bias (ppb)	3.65	5.14	5.14	5.86	3.34	5.95
Median_Bias (ppb)	3.64	4.99	5.32	6.02	3.73	5.95
Normalized_Mean_Bias_(%)	11.34	15.97	16.43	18.73	10.26	18.28
Normalized_Median_Bias_(%)	11.37	15.60	17.73	20.05	12.04	19.20
Coefficient_of_Determination_(R2)	0.61	0.61	0.60	0.60	0.60	0.63
Root_Mean_Square_Error	11.07	12.11	11.82	12.22	11.70	12.72
Index_of_Agreement	0.87	0.86	0.85	0.85	0.87	0.86
$PM_{2.5}$						
Mean_Bias (μg m^{-3})	1.93	4.28	2.72	3.55	-0.81	2.06
Median_Bias (μg m^{-3})	1.36	2.88	2.72	3.41	0.70	2.10
Normalized_Mean_Bias_(%)	22.45	49.76	32.13	41.87	-6.70	16.98
Normalized_Median_Bias_(%)	18.43	38.89	38.90	48.64	7.77	23.33
Coefficient_of_Determination_(R2)	0.05	0.01	0.03	0.01	0.01	0.02
Root_Mean_Square_Error	9.06	18.24	11.63	19.76	13.66	34.76
Index_of_Agreement	0.46	0.19	0.37	0.17	0.34	0.19

a. Statistics are calculated through a python-based diagnostic package MELODIES MONET (https://github.com/NOAA-CSL/MELODIES-MONET).

SI References

1. US Environmental Protection Agency, 2017 National Emissions Inventory (NEI), version 1. https://www.epa.gov/air-emissions-inventories/2017-national-emissions-inventory-nei-data, 2020.
2. T. Doumbia, C. Granier, N. Elguindi, I. Bouarar, S. Darras, G. Brasseur, B. Gaubert, Y. Liu, X. Shi, T. Stavrakou, S. Tilmes, F. Lacey, A. Deroubaix, T. Wang, Changes in global air pollutant emissions during the COVID-19 pandemic: a dataset for atmospheric modeling. *Earth Syst. Sci. Data* **13**, 4191–4206 (2021).
3. B. C. McDonald, Z. C. McBride, E. W. Martin, R. A. Harley, High-resolution mapping of motor vehiclecarbon dioxide emissions. *J. Geophys. Res.Atmos.* **119**, (2014).
4. B. C. McDonald, S. A. McKeen, Y. Y. Cui, R. Ahmadov, S.-W. Kim, G. J. Frost, I. B. Pollack, J. Peischl, T. B. Ryerson, J. S. Holloway, M. Graus, C. Warneke, J. B. Gilman, J. A. de Gouw, J. Kaiser, F. N. Keutsch, T. F. Hanisco, G. M. Wolfe, M. Trainer, Modeling ozone in the Eastern U.S. using a fuel-based mobile source emissions inventory. *Environ. Sci. Technol.* **52**, 7360-7370 (2018).
5. B. C. McDonald, D. R. Gentner, Long-term trends in motor vehicle emissions in u.s. urban areas. *Environ. Sci. Technol.* **47**, 10022-10031 (2013).
6. B. Hassler, B. C. McDonald, G. J. Frost, A. Borbon, D. C. Carslaw, K. Civerolo, C. Granier, P. S. Monks, S. Monks, D. D. Parrish, I. B. Pollack, K. H. Rosenlof, T. B. Ryerson, E. von Schneidemesser, M. Trainer, Analysis of long-term observations of NOx and CO in megacities and application to constraining emissions inventories. *Geophys. Res. Lett.* **43**, 9920–9930 (2016).
7. B. C. McDonald, T. R. Dallmann, E. W. Martin, R. A. Harley Long-term trends in nitrogen oxide emissions from motor vehicles at national, state, and air basin scales. *J. Geophys. Res. Atmos.* **117**, D00V18 (2012).
8. K. A. Yu, B. C. McDonald, R. A. Harley Evaluation of Nitrogen Oxide Emission Inventories and Trends for On-Road Gasoline and Diesel Vehicles. *Environ. Sci. Technol.* **55**, 6655–6664 (2021).
9. B. C. McDonald, J. A., de Gouw, J. B. Gilman, S. H. Jathar, A. Akherati, C. D. Cappa, J. L. Jimenez, J. Lee-Taylor, P. L. Hayes, S. A. McKeen, Y. Y. Cui, S.-W. Kim, D. R. Gentner, G. Isaacman-VanWertz, A. H. Goldstein, R. A Harley, G. J Frost, J. M. Roberts, T. B. Ryerson, M. Trainer, Volatile chemical products emerging as largest petrochemical source of urban organic emissions. *Science* **359**, 760-764 (2018).
10. H. S. Cao, D. K. Henze, K. Cady-Pereira, B. C. McDonald, C. Harkins, K. Sun, K. W. Bowman, T. M. Fu, M. O. Nawaz, COVID-19 lockdowns afford the first satellite-based confirmation that vehicles are an under-recognized source of urban NH3 pollution in Los Angeles. *Environ. Sci. Technol. Lett.* **9**, 3-9 (2022).
11. B. C. McDonald, A. H. Goldstein, R. A. Harley Long-term trends in California mobile source emissions and ambient concentrations of black carbon and organic aerosol. *Environ. Sci. Technol.* **49**, 5178-5188 (2015).
12. C. Harkins, B. C McDonald, D. K. Henze, C. Wiedinmyer, A fuel-based method for updating mobile source emissions during the COVID-19 pandemic. *Environ. Res. Lett.* **16**, (2021).

13. A. J. Kean, R. F. Sawyer, R. A. Harley A fuel-based assessment of off-road diesel engine emissions. *J. Air Waste Manag. Assoc.* **50**, 1929-1939 (2000).
14. *NONROAD2008a ModelRep.* 2010; Available from: https://www.epa.gov/moves/nonroad-technical-reports.
15. R. A. Harley, S. C. Coulter-Burke, T. S. Yeung Relating liquid fuel and headspace vapor composition for California reformulated gasoline samples containing ethanol. *Environ Sci Technol* **34**, 4088-4094 (2000).
16. S.-W. Kim, B. C. McDonald, S. Baidar, S. S. Brown, B. Dube, R. A. Ferrare, G. J. Frost, R. A. Harley, J. S. Holloway, H.-J. Lee, S. A. McKeen, J. A. Neuman, J. B. Nowak, H. Oetjen, I. Ortega, I. B. Pollack, J. M. Roberts, T. B. Ryerson, A. J. Scarino, C. J. Senff, R. Thalman, M. Trainer, R. Volkamer, N. Wagner, R. A. Washenfelder, E. Waxman, C. J. Young, Modeling the weekly cycle of NOx and CO emissions and their impacts on O3 in the Los Angeles South Coast Air Basin during the CalNex 2010 field campaign. *J. Geophys. Res. Atmos.* **121**, (2016).
17. M. M. Coggon, G. I. Gkatzelis, B. C. McDonald, J. B. Gilman, R. H. Schwantes, N. Abuhassan, K. C. Aikin, M. F. Arend, T. A. Berkoff, S. S. Brown, T. L. Campos, R. R. Dickerson, G. Gronoff, J. F. Hurley, G. Isaacman-VanWertz, A. R. Koss, M. Li, S. A. McKeen, F. Moshary, J. Peischl, V. Pospisilova, X. Ren, A. Wilson, Y. Wu, M.Trainer, C. Warneke, Volatile chemical product emissions enhance ozone and modulate urban chemistry,. *Proc. Natl. Acad. Sci. U.S.A.* **118**, e2026653118 (2021).
18. M. Li, B. C. McDonald, S. A. McKeen, H. Eskes, P. Levelt, C. Francoeur, C. Harkins, J. He, M. Barth, D. K. Henze, M. M. Bela, M. Trainer, J. A. de Gouw, G. J. Frost, Assessment of Updated Fuel-Based Emissions Inventories over the Contiguous United States using TROPOMI NO2 Retrievals. *J. Geophys. Res. Atmos.* **126**, e2021JD035484 (2021).
19. S.-W. Kim, B. C. McDonald, S. Seo, K.-M. Kim, M. Trainer Understanding the Paths of Surface Ozone Abatement in the Los Angeles Basin. *J. Geophys. Res. Atmos.* **127**, e2021JD035606 (2022).
20. C. E. Stockwell, M. M. Coggon, G. I. Gkatzelis, J. Ortega, B. C. McDonald, J. Peischl, K. Aikin, J. B. Gilman, M. Trainer, C. Warneke Volatile organic compound emissions from solvent- and water-borne coatings – compositional differences and tracer compound identifications. *Atmos. Chem. Phys.* **21**, 6005-6022 (2021).
21. G. I. Gkatzelis, M. M. Coggon, B. C. McDonald, J. Peischl, J B. Gilman, K. C. Aikin, M. A. Robinson, F. Canonaco, A. S. H. Prevot, M. Trainer, C. Warneke, Observations Confirm that Volatile Chemical Products Are a Major Source of Petrochemical Emissions in U.S. Cities. *Environ. Sci. Technol.* **55**, (2021).
22. M. M. Coggon, B. C. McDonald, A. Vlasenko, P. R. Veres, F. Bernard, A. R. Koss, B. Yuan, J. B. Gilman, J. Peischl, K. C. Aikin, J. DuRant, C. Warneke, S-M. Li, J. A. de Gouw, Diurnal variability and emission pattern of decamethylcyclopentasiloxane (D5) from the application of personal care products in two north American cities. *Environ. Sci. Technol.* **52**, 5610–5618 (2018).
23. A. M. Gorchov Negron, B. C. McDonald, S. A. McKeen, J. Peischl, R. Ahmadov, J. A. de Gouw, G. J. Frost, M. G. Hastings, I. B. Pollack, T. B. Ryerson, C.

Thompson, C. Warneke, M. Trainer, Development of a Fuel-Based Oil and Gas Inventory of Nitrogen Oxides Emissions. *Environ. Sci. Technol.* **52**, 10175–10185 (2018).

24. C. B. Francoeur, B. C. McDonald, J. B. Gilman, K. J. Zarzana, B. Dix, S. S. Brown, J. A. de Gouw, G. J. Frost, M. Li, S. A. McKeen, J. Peischl, I. B. Pollack, T. B. Ryerson, C. Thompson, C. Warneke, M.Trainer, Quantifying methane and ozone precursor emissions from oil and gas production regions across the contiguous US. *Environ. Sci. Technol.* **55**, 9129–9139 (2021).
25. J. Xing, J. Pleim, R. Mathur, G. Pouliot, C. Hogrefe, C. M. Gan, C. Wei, Historical gaseous and primary aerosol emissions in the United States from 1990 to 2010. *Atmos. Chem. Phys.* **13**, 7531–7549 (2013).
26. P. S. Rickly, M. M. Coggon, K. C. Aikin, R. J. Alvarez 2nd, S. Baidar, J. B. Gilman, G. I. Gkatzelis, C. Harkins, J. He, A. Lamplugh, A. O. Langford, B. C. McDonald, J. Peischl, M. A. Robinson, A. W. Rollins, R. H. Schwantes, C. J. Senff, C. Warneke, S. S. Brown, Influence of Wildfire on Urban Ozone: An Observationally Constrained Box Modeling Study at a Site in the Colorado Front Range. *Environ. Sci. Technol.* **57**, 1257–1267 (2023).
27. G. A. Grell, S. E. Peckham, R. Schmitz, S. A. McKeen, G. Frost, W. C. Skamarock, B. Eder, Fully coupled “online” chemistry within the WRF model. *Atmos. Environ.* **39**, 6957-6975 (2005).
28. P. O. Wennberg, K. H. Bates, J. D. Crounse, L. G. Dodson, R. C. McVay, L. A. Mertens, T. B. Nguyen, E. Praske, R. H. Schwantes, M. D. Smarte, J. M. St Clair, A. P. Teng, A. P., X. Zhang, J. H. Seinfeld, Gas-phase reactions of isoprene and its major oxidation products. *Chem. Rev.* **118**, 3337–3390 (2018).
29. C. Warneke, J. P. Schwarz, J. E. Dibb, O. Kalashnikova, G. Frost, J. Al-Saadi, S. S. Brown,W. A. Brewer,A. Soja, F. C. Seidel, R. A. Washenfelder, E. B. Wiggins, R. H. Moore, B. E. Anderson, C. Jordan, T. I. Yacovitch, S. C. Herndon, S. Liu, T. Kuwayama, D. Jaffe, N. Johnston, V. Selimovic, R. Yokelson, D. M. Giles, .B. N. Holben, P. Goloub, I. Popovici, M. Trainer, A. Kumar, R. B. Pierce, D. Fahey, J. Roberts, E. M. Gargulinski, D. A. Peterson, X. Ye, L. H. Thapa, P. E. Saide, C. H. Fite, C. D. Holmes, S. Wang, M. M. Coggon, Z. C. J. Decker, C. E. Stockwell, L. Xu, G. Gkatzelis, K. Aikin, B. Lefer, J. Kaspari,D. Griffin, L. Zeng, R. Weber, M. Hastings, J. Chai, G. M. Wolfe, T. F. Hanisco, J. Liao, P. Campuzano-Jost, H. Guo, J. L. Jimenez, J. Crawford, Fire Influence on Regional to Global Environments and Air Quality (FIREX-AQ). *J. Geophys. Res.-Atmos.* **128**, e2022JD037758 (2023).
30. X. Jin, A. Fiore, K. F. Boersma, I. De Smedt, L. Valin, Inferring Changes in Summertime Surface Ozone−NOx−VOC Chemistry over U.S. Urban Areas from Two Decades of Satellite and Ground-Based Observations. *Environ. Sci. Technol.* **54**, 6518-6529 (2020).
